# Design, green synthesis and biological evaluation of fluorinated *N*-acyl sulfonamides as novel anti-inflammatory agents: an *in vivo* and *in silico* study

**DOI:** 10.1039/d6ra01380e

**Published:** 2026-03-23

**Authors:** Zaineb Litim, Ameni Ben Abdeljaoued, Amal Abdelhamid, Abderrahman Bouraoui, İsmail Özdemir, Naceur Hamdi, Mohamed Ali Soussi, Jamil Kraiem

**Affiliations:** a Laboratoire de Développement Chimique, Galénique et Pharmacologique des Médicaments (LR12ES09), Faculté de Pharmacie de Monastir, Université de Monastir Rue Avicenne 5000 Monastir Tunisia jamil.kraiem@fphm.u-monastir.tn; b Inonu University, Faculty of Arts and Sciences, Department of Chemistry Malatya 44280 Turkey; c Research Laboratory of Environmental Sciences and Technologies (LR16ES09), Higher Institute of Environmental Sciences and Technology, University of Carthage Hammam-Lif Tunisia

## Abstract

The *N*-acylsulfonamide functional group constitutes a key moiety in numerous successful drugs, primarily due to its high chemical stability and favorable human tolerance profile. Herein, we report the development of a new eco-friendly and highly efficient approach for the preparation of *N*-acylsulfonamides. This method involves the synthesis of *N*-sulfonyloxaziridines *via* oxidation of the corresponding *N*-sulfonylimines, followed by a facile rearrangement into *N*-acylsulfonamides under mild and green conditions. The methodology afforded a diverse range of *N*-acylsulfonamides in good yields and high atom economy (AE). The synthesized compounds were subsequently evaluated for their *in vivo* anti-inflammatory activity. Specifically, the fluorinated *N*-acylsulfonamides 3c, 3e, and 3f exhibited potent inhibitory activity against carrageenan-induced inflammation, surpassing the reference drug (diclofenac). Analysis of the structure-activity relationship (SAR) highlighted the critical role of fluorine in enhancing this anti-inflammatory potential. Molecular docking and ADME-T studies were undertaken to elucidate the molecular mechanisms of action and predict the pharmacokinetic profile of these compounds on their biological targets.

## Abstract 2

A green synthesis of *N*-acylsulfonamides was developed. Some of these compounds showed *in vivo* anti-inflammatory activity that surpasses the efficacy of diclofenac. These results are in good agreement with the *in silico* study.

## Graphical abstract

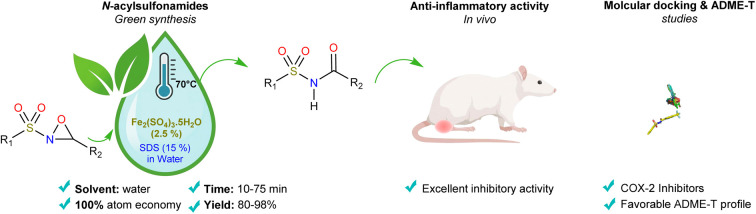

## Introduction

Inflammatory diseases and chronic inflammation play a central role in global morbidity and mortality, particularly through their involvement in cardiovascular disease, asthma, rheumatoid arthritis, type 2 diabetes, and certain cancers.^1,2^ Non-steroidal anti-inflammatory drugs (NSAIDs) are the most commonly used to relieve pain and signs of inflammation associated with inflammatory disorders. They inhibit cyclooxygenase (COX) enzymes and thus block the biosynthesis of prostaglandins (PG) from arachidonic acid. However, most NSAIDs frequently cause various side effects, sometimes leading to the withdrawal of some of them from the market.^3,4^ Hence, the search for new powerful anti-inflammatory molecules with an improved safety profile remains a major challenge.

*N*-Sulfonamide and its derivatives have long received considerable attention as an important class of small molecules with high medicinal value.^5,6^ This group was considered also as an ideal bioisostere of carboxylic acids due to its similar acidity (p*K*_a_ range of 3.5–4.5) and ability to form a similar network of H-bond, while also offering enhanced stability to enzymatic and chemical hydrolysis.^7–9^ Over the past few years, numerous structures containing *N*-sulfonamide moiety have been designed and synthesized with broad spectrum of pharmacological activities.^10–14^

Fluorine atom, characterized with its small atomic size and high electronegativity, gives organic molecules specific physicochemical and biological properties. In fact, integrating this atom into a molecule makes it possible to modulate its lipophilicity, metabolic stability and affinity for pharmacological receptors. Indeed, the introduction of fluorine has become an essential strategy in the design of new molecules with potential therapeutic effects, offering new prospects for the development of more effective and selective drugs.^15–18^

From a synthetic perspective, a series of methods to prepare *N*-acylsulfonamide and their derivatives have been developed. Historically, the most common used methods involved either the sulfonylation of amides^19–21^ or the acylation of sulfonamides with acylating agent such as carboxylic acid,^22–27^*N*-acylbenzotriazole,^28^ ester,^29,30^ acid chloride or carboxylic anhydride.^31–34^ These synthetic routes generally work well; however, they often suffer from one or more of the following disadvantages: harsh reaction conditions, low yields, use of stoichiometric quantities of coupling agents and bases, and generation of excessive waste which can be difficult to remove. Alternative methodologies described the synthesis of *N*-acylsulfonamides from sulfonylazides^35,36^ and amidation of aldehydes.^37–39^ Sulfonylisocyanates have been also employed as starting material. However, their hydrolytic instability leads to considerable handling issues and general lack of commercially available derivatives.^40,41^ Novel routes involving the transacylation of *N*-acylsulfonamides have been reported, enabling the creation of diverse analogues from a single *N*-acylsulfonamide.^42,43^ More recently, Lam *et al.* have developed a transition-metal-free photocatalytic S–N coupling reaction for *N*-acylsulfonamide synthesis employing hydroxamic acid and sodium organosulfinate.^44^ Despite these advancements, we think that developing an efficient, eco-friendly, and more atom-economic methodology to access to this ubiquitous class would be highly interesting.

Transition metal-catalyzed rearrangement of oxaziridines constitutes a powerful and well-established method for amide synthesis. In our previous work, we have developed an efficient approach for the synthesis of *N*-alkylamides *via* the rearrangement of the corresponding *N*-alkyloxaziridines in water as the solvent and iron sulfate (2.5 mol%) as the catalyst.^45^ This approach offers the advantage of total atom-economy, a fundamental and crucial asset in the field of green chemistry. Similarly, the rearrangement of *N*-sulfonyloxaziridines appears to be a promising synthetic route to *N*-acylsulfonamides. In this context, and in line with our interest in the synthesis and application of *N*-sulfonylimines and *N*-sulfonyloxaziridines in organic synthesis,^46,47^ we propose an eco-friendly methodology to access to *N*-acylsulfonamides, which involves the synthesis of *N*-sulfonyloxaziridines by oxidizing the corresponding *N*-sulfonylimines, followed by their rearrangement to *N*-acylsulfonamides under mild and green conditions. We chose to introduce one or more fluorine atoms into the structure of some *N*-acylsulfonamides. We hypothesized that fluorinated *N*-acylsulfonamides could exhibit an improved anti-inflammatory activity and a favorable pharmacological profile. The synthesized compounds were evaluated for their *in vivo* anti-inflammatory activity. Furthermore, a docking study was conducted against COX-2 to better understand the interactions of these molecules within the binding site.

## Results and discussion

### Chemistry

**Scheme 1:**
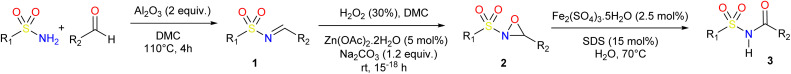
Green synthesis of *N*-acylsulfonamides from sulfonamides and aldehydes.

To investigate the rearrangement of *N*-sulfonyloxaziridines to *N*-acylsulfonamides, we adopted an eco-friendly and efficient approach. The synthetic procedure was performed in three-step sequence as illustrated in Scheme 1.

At first, we prepared *N*-sulfonylimines 1*via* the condensation of sulfonamides with aromatic aldehydes.^46^ We used dimethyl carbonate (DMC) as a green solvent and neutral alumina as recyclable dehydrating agent. Second, the oxidation of *N*-sulfonylimines was carried out at room temperature using an eco-friendly oxidant system consisting of DMC and H_2_O_2_ in the presence of sodium carbonate and a catalytic amount of Zn(OAc)_2_·2H_2_O.^47^ Thereafter, we explored the rearrangement of the synthesized *N*-sulfonyloxaziridines 2 to the corresponding *N*-acylsulfonamides 3. We followed the procedure previously developed by our group for the rearrangement of *N*-alkyloxaziridines.^45^ In this study, we chose water as the solvent of the reaction, replacing volatile and flammable organic solvents.^48,49^ This choice aligned with our aim to promote economical, safe, and environmentally friendly organic transformations. In addition, we explored the use of iron-based catalyst because of their widespread availability, low cost, and reduced toxicity profile. A catalytic amount of SDS was employed to improve the solvation of organic reagents in aqueous media.^45,50–52^

We applied the optimized reaction conditions previously described [0.5 mmol of oxaziridine, 2.5 mol% Fe_2_(SO_4_)_3_·5H_2_O, 15% SDS, 1 mL H_2_O, 70 °C]^45^ to the rearrangement of a variety of *N*-sulfonyloxaziridines (Table 1). The reaction was continuously stirred until disappearance of the oxaziridine. Interestingly, a total conversion to the corresponding amide 3 was observed within 10–75 minutes. The crude mixture was then extracted with ethyl acetate, and followed by simple recrystallization to yield pure product. The results summarized in Table 1 show the excellent compatibility of the reaction with a wide variety of substrates (Table 1). The corresponding *N*-acylsulfonamides 3a–j were obtained in good to excellent yields (80–98%). Furthermore, the variability of the reaction times given in Table 1 revealed a correlation between the reactivity of the oxaziridine during the rearrangement step and the nature of its substituents. Indeed, the presence of electron-withdrawing groups, namely the trifluoromethyl group, on the aromatic ring of 3-aryloxaziridine (3f, 3g, 3j) appears to slow down the reaction. The originality of this approach lies in its ability to ensure total conversion of oxaziridines to amides without formation of by-products. This aspect is particularly attractive in the context of green chemistry.

**Table 1:** Synthesis of *N*-acylsulfonamide derivatives 3a–j*via* the rearrangement of *N*-sulfonyloxaziridines^a^

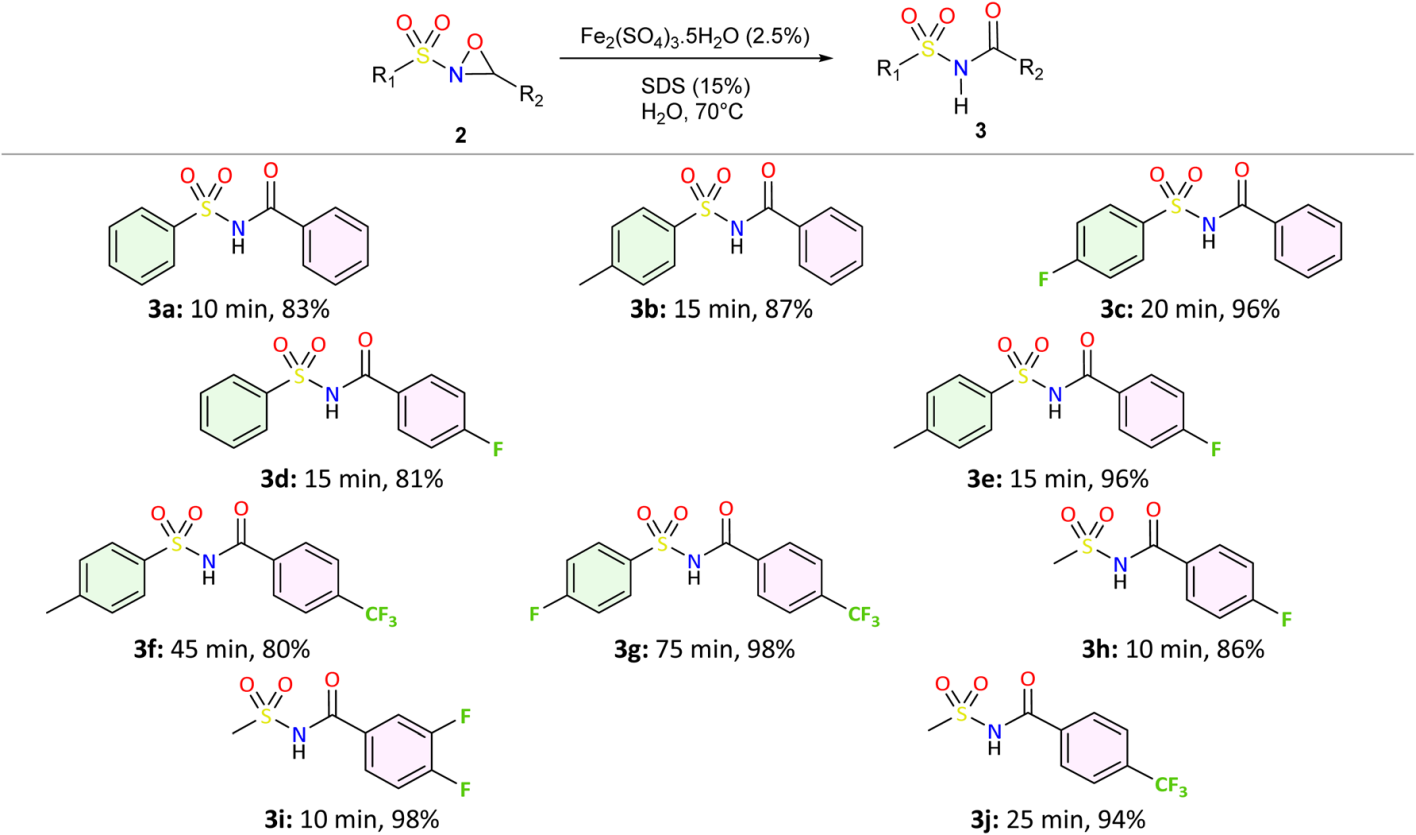

a Reaction conditions: *N*-sulfonyloxaziridine (0.5 mmol), Fe_2_(SO_4_)3·5H_2_O (2.5 mol%), SDS (15%), H_2_O (1 mL), 70 °C.^47^ The mixture was stirred until disappearance of oxaziridine (TLC).

**Scheme 2:**
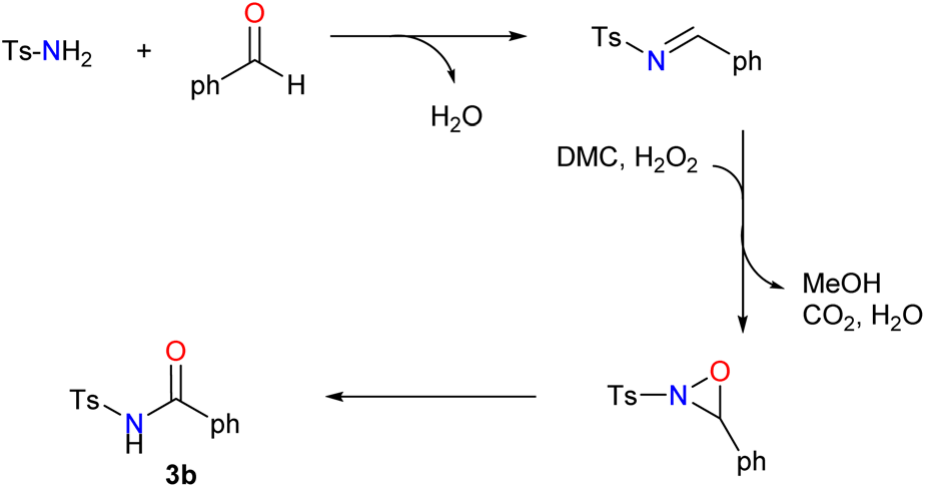
Synthetic approach to *N*-acylsulfonamides for atom economy analysis.

From an economic and an environmental point of views, our overall approach, starting from sulfonamides and aldehydes and involving sulfonylimines and sulfonyloxaziridines as intermediates, appears to be greener and more cost-effective than the classic routes described in the literature for several reasons: (i) we used only green solvents such as DMC and water, while other methods generally used toxic solvents. (ii) The catalysts and additives used are effective, inexpensive and ecologically benign. (iii) Process-generated waste must be taken into consideration in the design of environmentally sustainable chemical processes. Our method generates only water, CO_2_ and methanol as byproducts in stoichiometric quantities, thereby minimizing the environmental impact (Scheme 2). (iv) The developed method is highly efficient: by maximizing the incorporation of reactant atoms into the final product, a high atom economy (AE) was attained, combined with excellent yields. For example, as shown in Scheme 2, the three-step synthesis of *N*-acylsulfonamide 3b demonstrated an atom economy of 69%. In essence, the green aspects, high yields, and simple handling make this methodology an attractive and practical alternative to the synthesis of *N*-acylsulfonamides.

### *In vivo* anti-inflammatory activity

**Fig. 1:**
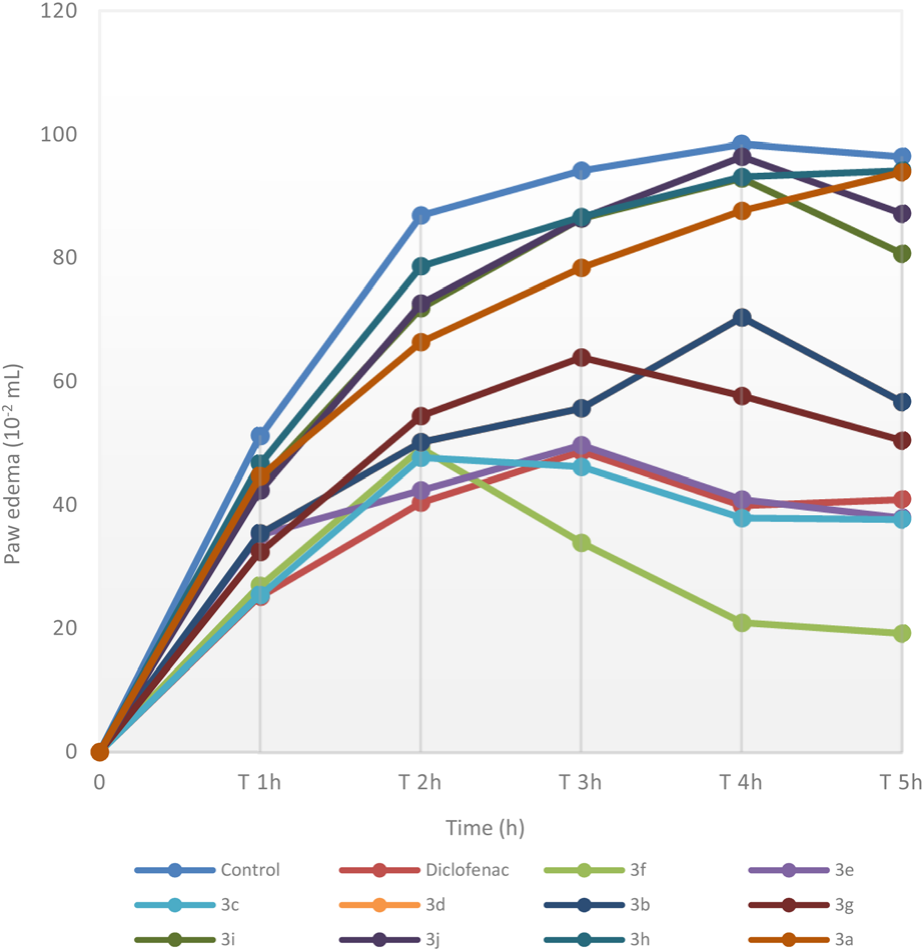
Effect of intraperitoneal administration of *N*-acylsulfonamides derivatives 3a–j on the carrageenan-induced paw edema in comparison to the reference group (diclofenac).

The current study is undertaken in aim to screen and evaluate the anti-inflammatory efficacy of a new series of *N*-acylsulfonamides 3a–j in experimental rat model. The results of the carrageenan-induced paw edema are given in Table 2 and Fig. 1. The injection of carrageenan suspension to the control group resulted in a time-dependent increase in paw edema. However, the pre-treatment with diclofenac used as a reference drug at the dose of 25 mg kg^−1^ significantly prevented the carrageenan effect with a 48.28% and 57.51% reduction in edema at the 3rd and 5th h, respectively. *N*-acylsulfonamide 3f exhibited the higher anti-inflammatory activity, surpassing the reference drug with inhibition percentages of 62.22% and 80.15% at the 3rd and 5th h respectively. Although, *N*-acylsulfonamide derivatives 3c and 3e displayed higher anti-inflammatory potential than that exerted by diclofenac with a reduction of 61.08% and 60.82% in paw volume, respectively, 5 h after carrageenan injection. The pre-treatment with compounds 3d and 3g showed a moderate activity. However, the rest of the compounds of the series did not exhibit considerable anti-inflammatory activity in this model, even after 5 hours of experimentation. The results of the anti-inflammatory activity of compounds 3a–j revealed a significant structure-activity relationship. As it can be seen, *N*-methylsulfonylbenzamides 3h, 3i and 3j were less active than *N*-arylsulfonylbenzamides 3d, 3e, 3f and 3g. In fact, the replacement of the aryl group on R with a methyl group reduced the anti-inflammatory activity. This may be attributed to the contribution of the phenyl ring to the pharmacokinetic properties of *N*-acylsulfonamides as well as their interaction with pharmacological targets involved in the inflammatory process.

**Table 2:** Anti-inflammatory effect of the intraperitoneal administration of *N*-acylsulfonamides derivatives 3a–j and of reference drug (diclofenac) in carrageenan-induced rat paw edema test

Samples	Dose (mg kg^−1^)	Paw edema volume (10^−2^ mL)			% Of inhibition of paw edema		
		1 h	3 h	5 h	1 h	3 h	5 h
Control	—	51.25 ± 3.38	94.25 ± 1.25	96.50 ± 1.50	—	—	—
Diclofenac	25	25.25 ± 1.88	48.75 ± 3.25	41.00 ± 3.50	50.73	**48.28**	**57.51**
3a	50	44.75 ± 3.75	78.5 ± 3.75	94.00 ± 3.50	12.68	12.78	3.50
3b	50	35.50 ± 2.50	71.25 ± 4.25	87.50 ± 3.50	30.73	20.83	9.79
3c	50	25.50 ± 3.00	46.25 ± 2.75	37.75 ± 3.88	50.24	**48.61**	**61.08**
3d	50	35.50 ± 2.00	55.75 ± 3.88	56.75 ± 3.25	30.73	38.06	41.49
3e	50	35.25 ± 1.88	49.75 ± 4.25	38.00 ± 2.50	31.22	**44.72**	**60.82**
3f	50	27.00 ± 2.50	34.00 ± 1.00	19.25 ± 1.88	47.32	**62.22**	**80.15**
3g	50	32.50 ± 2.00	48.75 ± 3.25	41.00 ± 3.50	36.59	28.89	47.94
3h	50	46.75 ± 5.25	86.75 ± 3.75	94.25 ± 6.12	8.78	3.61	2.84
3i	50	44.00 ± 4.00	86.50 ± 3.00	80.75 ± 3.50	14.15	3.89	16.75
3j	50	42.50 ± 1.00	86.50 ± 2.00	87.25 ± 2.25	17.07	3.89	10.05

Furthermore, compounds 3c–g revealed a significant anti-inflammatory activity when compared to their structural analogues without fluorine (3a and 3b). More specifically, *N*-acylsulfonamides containing tow phenyl rings and at least one of them substituted with a fluorine atom or trifluoromethyl group are the best anti-inflammatory agents. This fact is unsurprising since introduction of a fluorine atom into drug molecules is a well-established strategy in medicinal chemistry, particularly through improved efficacy and selectivity (*e.g.*, selective COX-2 inhibitors (celecoxib) and fluoroquinolones). This improvement is primarily due to the unique chemical properties of fluorine, namely small atomic size and high electronegativity, which influence various physicochemical and pharmacokinetic properties of drugs.^15–18,53^ On the other hand, *N*-acylsulfonamide 3e showed an anti-inflammatory effect better than its structural analogue 3d. Indeed, introducing a methyl group in *para*-position of the phenyl ring on R increased clearly the activity, suggesting a significant effect of this group on the anti-inflammatory potential.

### Molecular docking

To investigate the COX-II-mediated anti-inflammatory effect of our synthesized *N*-acylsulfonamide compounds, molecular docking simulations were performed using the human cyclo-oxygenase enzyme COX-2 as the target. X-ray crystal structures of COX-2 (PDB ID: 5kir) were obtained from the Protein Data Bank (https://www.rcsb.org/). Given the protein's homodimeric nature, only one chain (Chain A) was retained and prepared by removing heteroatoms and water molecules while adding polar hydrogens.

The 3D conformations of the *N*-acylsulfonamide compounds were generated using the online cactus service provided by the National Cancer Institute (https://cactus.nci.nih.gov/). AutoDock tools was used to add polar hydrogens, compute Gasteiger charges, and define rotatable bonds. The docking grid was set to encompass the well-established active site pocket containing ARG120, GLN192, TYR385, ARG513, SER530, and VAL523, known for interactions with multiple anti-inflammatory drugs.^54–56^

The stability of the protein-ligand complexes was assessed by calculating the scoring function using AutoDock Vina 1.2.0,^57^ with a rigid protein model. Interactions were analyzed using AutoDock Tools,^58^ the Protein-Ligand Interaction Profiler^59^ (PLIP) and Protein Plus, and UCSF Chimera 1.18.^60^ Diclofenac was docked under identical conditions for comparative purposes.

**Fig. 2:**
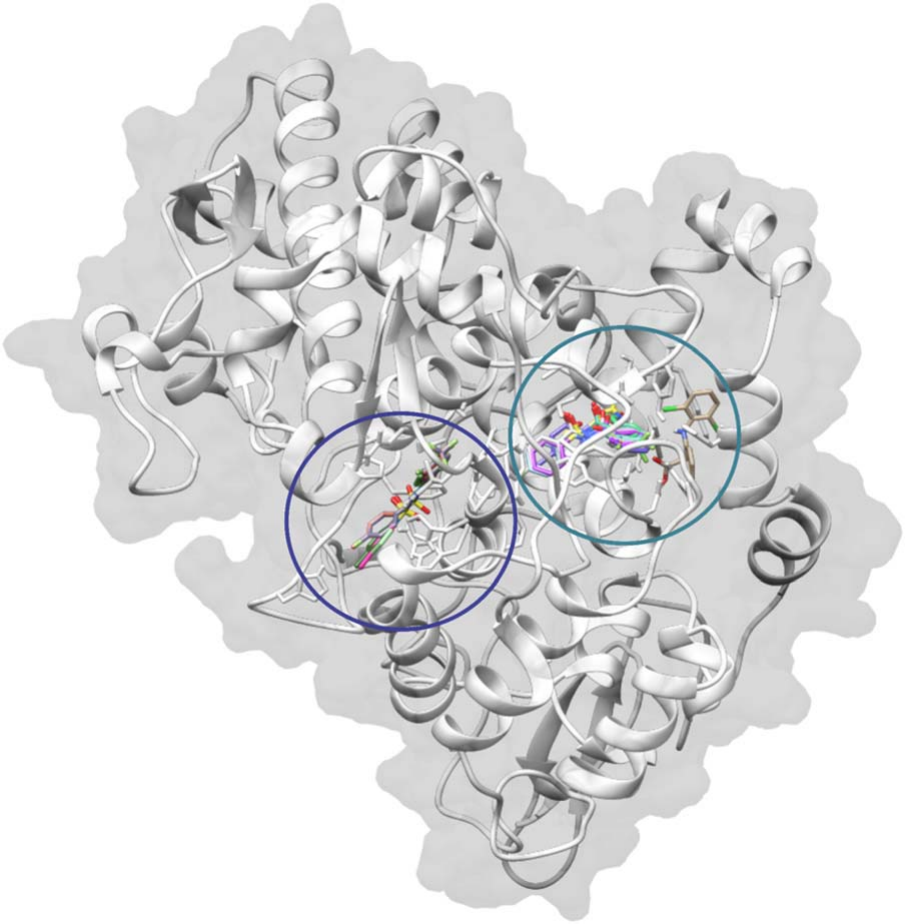
Binding modes of docked ligands in COX-2 (PDB: 5KIR) exhibiting two distinguished modes: Mode 1 (green blue) and Mode 2 (dark blue).

Table 3 summarizes the docking scores and binding modes identified through rigid site-specific docking. Two distinct binding behaviors were observed. Most compounds exhibited both binding modes: Mode 1, where the molecule binds in the central catalytic pocket, interacting with key residues such as PHE518, TRP387, and LEU352 (Fig. 2 green blue)—which are conserved in human COX-I and COX-II, and thus non-specific; and Mode 2, where the molecule occupies a secondary site adjacent to the catalytic pocket (Fig. 1 dark blue). This Mode 2 region aligns with an extended pocket noted in earlier computational studies.^61,62^

**Table 3:** Binding modes and scores (kcal mol^−1^)

		Mode 1		Mode 2	
	Molecule	Score	Interactions	Score	Interactions
	Diclofenac	−6.6	Hydrophobic: PRO86, VAL89, LEU93, VAL116, H-bond: SER119, π-stacking interaction: ARG120		No interaction reported within threshold
3a	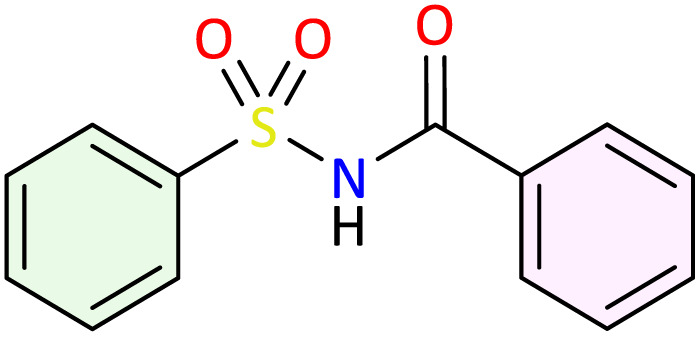	−8.1	Hydrophobic: LEU352, TRP387, PHE518		No interaction reported within threshold
3b	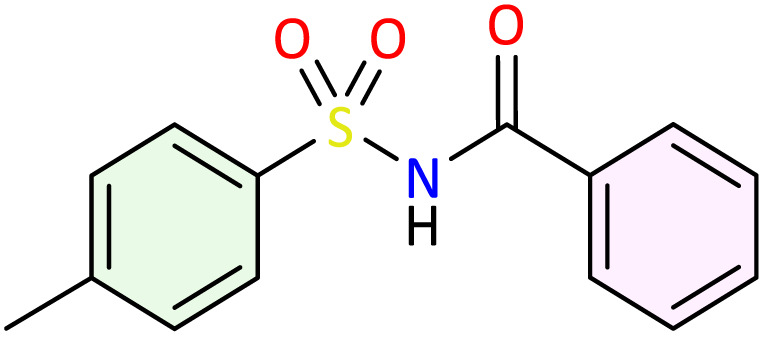	−8.8	Hydrophobic: LEU352, TRP387, PHE518	−8.0	Hydrophobic: ALA202, GLN203, TYR385, H-bond: ASN382, HIS386, π-stacking interaction: HIS386
3c	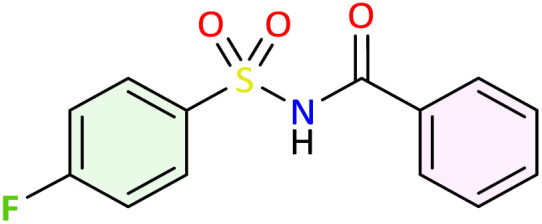	−8.7	Hydrophobic: LEU384, TRP387, TYR385, PHE518		No interaction reported within threshold
3d	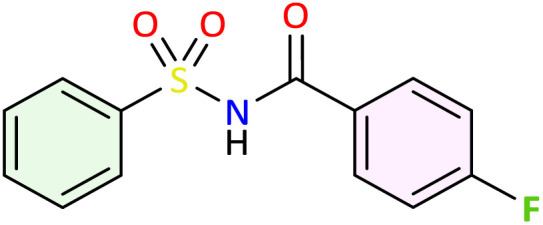	−8.4	Hydrophobic: LEU384, TYR385, PHE518	−7.1	Hydrophobic: ALA202, GLN203, TYR385, H-bond: ASN382, π-stacking interaction: HIS386
3e	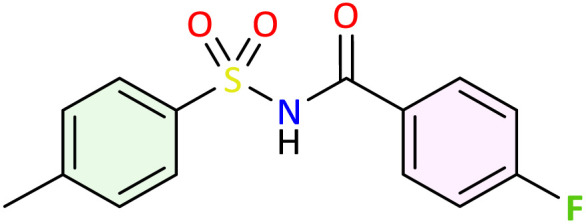	−9.1	Hydrophobic: LEU352, TRP387, PHE518	−8.7	Hydrophobic: ALA202, GLN203, TYR385, H-bond: ASN382, π-stacking interaction: HIS386
3f	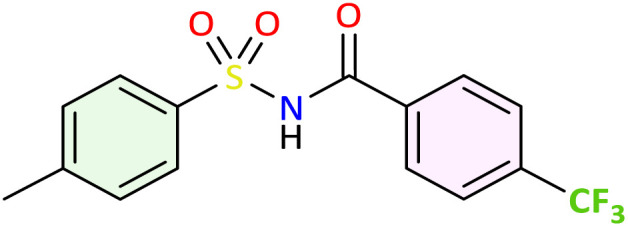		No interaction reported within threshold	−9.0	Hydrophobic: ALA202, GLN203, PHE210, π-stacking interaction: GLN203 (Fig. 3)
3g	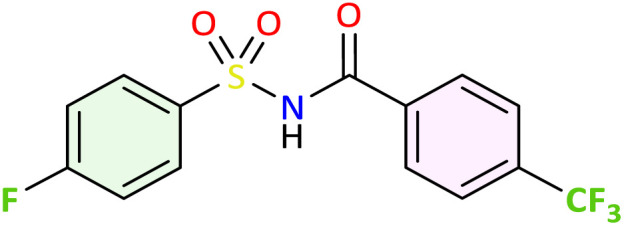		No interaction reported within threshold	−8.9	Hydrophobic: ALA202, GLN203, PHE210, halogen-bonds: GLN203
3h	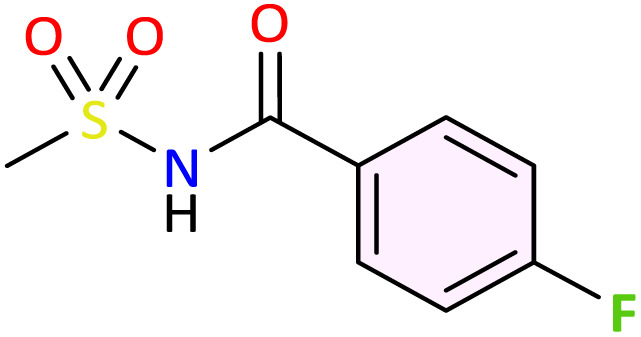	−6.8	Hydrophobic: PHE518, VAL523	−6.8	Hydrophobic: ALA202, GLN203, TYR385, H-bond: ASN382
3i	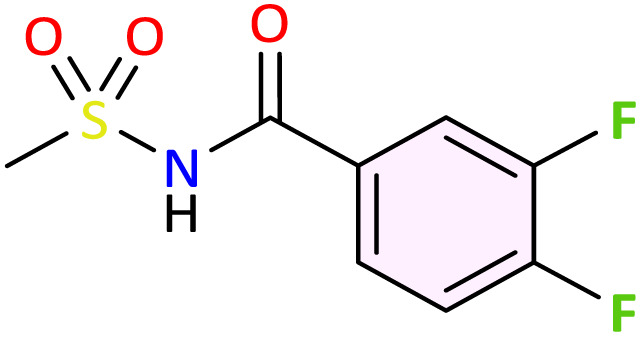	−7.2	Hydrophobic: VAL523, PHE518	−7.2	Hydrophobic: ALA202, GLN203, TYR385, H-bond: ASN382
3j	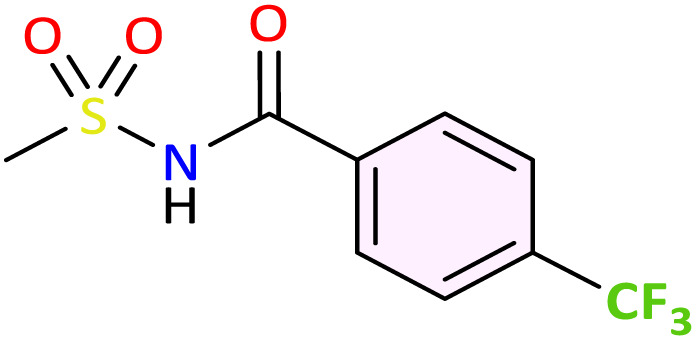	−8.1	Hydrophobic: TYR355, PHE518, VAL523, H-bond: ARG120, TYR355	−7.9	Hydrophobic: GLN203, H-bond: ASN382

The secondary site (Mode 2) demonstrates an enhanced capacity to accommodate bulky substituents. This results from the larger accessible volume in this region of the COX-2 binding site, which is approximately 25% more voluminous than the corresponding region in COX-1. This enlargement is primarily due to the Val509 substitution (Ile523 in COX-1) and the conformational flexibility of residue Leu531. Structurally and according to this X-ray crystallography structure (PDB id: 5KIR), Modes 1 and 2 represent different orientations within a continuous binding region, separated and defined by the side chains of these residues.^63,64^

**Fig. 3:**
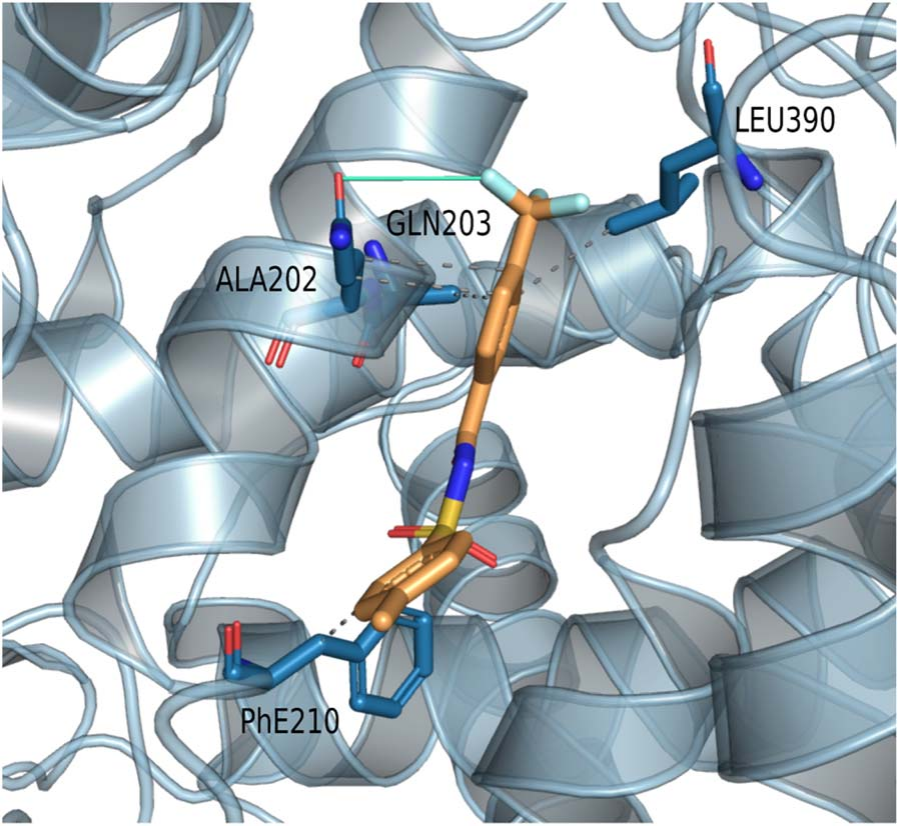
3D view of the binding of 3f (in binding Mode 2, generated using PLIP) showing mainly interactions with polar residues GLN203 and PHE210, a key characteristic of the second pocket (binding Mode 2): dashed gray: hydrophobic, solid light green: halogen bond.

*N*-acylsulfonamide 3f, the most active compound *in vivo*, displayed a unique and strong preference for Mode 2, with a high docking score of −9.0 kcal mol^−1^. Its binding pose is stabilized within the peroxidase site *via* hydrophobic interactions (ALA202, GLN203, PHE210) and a distinctive interaction with GLN203 (Fig. 3). The Mode 2 pocket, provides sufficient spatial accommodation for sterically demanding groups (like CF3 in 3f and 3g), explaining the preferential binding of compounds in this region rather than the more constrained Mode 1 site. 3g also exhibited a preference for Mode 2, achieving a comparable docking score of −8.9 (kcal mol^−1^) while having less efficiency *in vivo* compared to 3f, highlighting the limits of rigid anchoring in the explanation of the differences in efficiency. Compounds such as 3b, 3d, 3e and 3i show strong binding in both modes, indicating a versatile dual-site interaction profile with COX-I and COX-II (Fig. 2, Table 3).

Notably, 3a and 3b, despite achieving high docking scores in Mode 1 based predominantly on hydrophobic interactions (*e.g.*, with LEU352, TRP387), exhibited insufficient inhibitory effects in biological assays. This discrepancy highlights that while hydrophobic interactions contribute to binding, they may be insufficient for effective inhibition, especially if the mechanism is a competitive one with the biological ligand-arachidonic acid. The critical lack of a key interaction with ARG120, which is essential for anchoring classic acidic NSAIDs,^63^ likely prevented them from effectively competing with the physiological substrate.

### ADME-T screening

We also conducted a quick ADME-Tox screening using a web-based service (ADME-T-AI) with models built using more than 40 ADME-T databases and a graph neural network architecture (Chemprop-RDKit). Table 4 presents ADMET properties predicted by ADMET-AI, where physicochemical descriptors such as log *P* (octanol/water partition coefficient), H-bond donors/acceptors, TPSA (topological polar surface area), and QED (quantitative estimate of drug-likeness) align with Lipinski's rule of five criteria for oral drug-likeness.

**Table 4:** ADMET profile of *N*-acylsulfonamide derivatives 3a–j

Parameters	3a	3b	3c	3d	3e	3f	3g	3h	3i	3j	Diclofenac
**Physicochemical proprieties**											
Molecular weight (g mol^−1^)	261.30	275.33	279.29	279.29	293.32	343.33	347.29	217.22	235.21	267.23	296.15
Log *P* (log-ratio)	1.81	2.11	1.94	1.94	2.25	3.13	2.96	0.52	0.65	1.39	4.36
H-bond acceptors	3	3	3	3	3	3	3	3	3	3	2
H-bond receptors	1	1	1	1	1	1	1	1	1	1	2
Druglikeness (QED)	0.92	0.93	0.93	0.93	0.94	0.93	0.87	0.78	0.82	0.88	0.88
TPSA (Å^2^)	63.24	63.24	63.24	63.24	63.24	63.24	63.24	63.24	63.24	63.24	49.33

**Absorption**											
HIA	1.00	1.00	1.00	1.00	1.00	1.00	1.00	1.00	1.00	1.00	1.00
Oral bioavailability	0.92	0.95	0.96	0.96	0.96	0.95	0.96	0.96	0.98	0.96	0.96
Aqueous solubility (log(mol L^−1^)	−2.60	−3.07	−3.11	−3.22	−3.42	−3.93	−4.01	−1.82	−1.73	−2.16	−4.37
Lipophilicity (log-ratio)	0.49	0.58	1.02	1.24	1.18	1.86	2.16	−0.62	−0.39	0.69	1.14

**Distribution**											
BBB permeability	0.72	0.67	0.81	0.81	0.77	0.81	0.81	0.93	0.95	0.92	0.61
PPBR (%)	89.64	96.01	92.53	93.83	97.12	100.00	100.00	65.05	65.52	83.87	96.62

**Metabolism**											
CYP2C9	0.67	0.75	0.73	0.75	0.76	0.71	0.69	0.48	0.56	0.45	0.65
CYP2D6	0.03	0.05	0.06	0.06	0.08	0.13	0.14	0.07	0.07	0.10	0.03
CYP3A4	0.16	0.22	0.25	0.25	0.32	0.39	0.38	0.30	0.33	0.36	0.42

**Excretion**											
Half-life (hr)	25.01	21.74	26.00	21.38	37.54	31.64	54.41	0.00	0.00	0.00	0.00

**Toxicity**											
hERG blocking	0.01	0.03	0.05	0.05	0.10	0.35	0.37	0.03	0.03	0.10	0.05
Mutagenicity	0.004	0.01	0.01	0.01	0.02	0.02	0.01	0.04	0.03	0.03	0.07
Liver injury	0.97	0.97	0.98	0.98	0.97	0.96	0.97	0.98	0.98	0.96	0.93
Carcinogenicity	0.57	0.67	0.65	0.66	0.74	0.86	0.83	0.43	0.41	0.72	0.51

Absorption parameters show high HIA (human intestinal absorption) and oral bioavailability probabilities (∼1.0), supported by favorable solubility and lipophilicity profiles that favor good oral exposure, while distribution metrics indicate strong PPBR (plasma protein binding ratio, 65–100%) and BBB (blood–brain barrier) permeability (0.67–0.95), beneficial for cerebral edema treatment but necessitating neurotoxicity evaluation. Metabolism predictions identify primary CYP2C9 substrate (probability ranging from 0.45 to 0.76) with low CYP2D6/CYP3A4 involvement and extended half-lives for bulkier analogs (3e–3g), while toxicity flags high drug-induced liver injury risk (0.93–0.98) attributable to sulfonamide features alongside low hERG blockade and mutagenicity.

The subtle lipophilicity/clearance differences might explain efficacy shifts between 3f and 3g inhibition in Table 2 despite similar binding reflected by the rigid docking especially that for 3g, the inhibition exhibited with inhibition percentage of paw edema if 3f was fluctuating (up, down then up) and not linearly incrementing as observed with 3g and 3e.

Complementary off-target screening *via* ProTox 3.0 identified binding predictions beyond the intended COX-2 target. Prostaglandin G/H synthase 1 (COX-1) appeared as a predicted target for compounds 3a, 3b, 3c, 3d, 3e, 3f and 3g, suggesting potential dual COX inhibition that would be beneficial to avoid cardiotoxicity but raises gastrointestinal safety concerns. More surprisingly, opioid receptor mu (µ) binding was predicted for 3a, 3b and 3f that could contribute to analgesic properties complementing anti-inflammatory activity.

## Conclusion

Herein, we have developed a green and highly efficient synthesis approach to access to *N*-acylsulfonamides. Indeed, *N*-sulfonyloxaziridines, prepared from *N*-sulfonylimines, underwent a catalytic rearrangement mediated by iron(iii) in water as the solvent, affording the corresponding *N*-acylsulfonamides in high yields (up to 98%). The advantage of this method lies in its ability to ensure total conversion of oxaziridines to the corresponding amides, without the formation of by-products, thus, guaranteeing 100% atomic economy. Thereafter, the synthesized *N*-acylsulfonamides 3a–j were screened for their *in vivo* anti-inflammatory activity. The preliminary results obtained were encouraging, namely, for the fluorinated compounds 3c, 3e and 3f. These molecules displayed excellent inhibitory activity against carrageenan-induced inflammation, surpassing the efficacy of the reference drug (diclofenac). Although, a comprehensive analysis integrating molecular docking and ADME-T predictions confirmed that the *N*-acylsulfonamide derivatives yielded high docking scores and maintained favorable ADME-T profiles. The results obtained in this study confirm the initial hypothesis that fluorinated *N*-acylsulfonamides could be effective therapeutic candidates and thus warrant further investigation.

## Ethical Statement

All animal procedures were performed according to the guidelines established by the European Union regarding the Use and the Animal Care (CCE Council 86/609) and with the approval of the ethic committee on the research in life sciences and health of the Higher Institute of Biotechnology of Monastir (University of Monastir, Tunisia).

## Conflicts of interest

“There are no conflicts to declare”.

## Supplementary Material

RA-016-D6RA01380E-s001

## Data Availability

The data supporting this article have been included as part of the supplementary information (SI). Supplementary information: experimental procedures and copies of NMR spectra. See DOI: https://doi.org/10.1039/d6ra01380e.
