# Supplementary material for: Design, green synthesis and biological evaluation of fluorinated N-acyl sulfonamides as novel anti-inflammatory agents: an in vivo and in silico study
Source: RSC Adv. 2026 Mar 23;16(18):15992–6000. doi: 10.1039/d6ra01380e (PMC13006991; doi:10.1039/d6ra01380e)
Supplement: RA-016-D6RA01380E-s001 [file RA-016-D6RA01380E-s001.pdf]

Supplementary Information (SI) for RSC Advances.
This journal is © The Royal Society of Chemistry 2026

Design, Green Synthesis and Biological Evaluation of Fluorinated *N*-Acylsulfonamides as Novel Anti-Inflammatory Agents: An *In Vivo* and *In Silico* Study

Zaineb Litim,*[a] Ameni Ben Abdeljaoued,[a] Amal Abdelhamid,[a] Abderrahman Bouraoui, İsmail Özdemir,[b] Naceur Hamdi,[c] Mohamed Ali Soussi[a] and Jamil Kraiem*[a]

[a] *Laboratoire de Développement Chimique, Galénique et Pharmacologique des Médicament (LR12ES09)s, Faculté de Pharmacie de Monastir, Université de Monastir, Rue Avicenne, 5000, Monastir Tunisia. E-mail: jamil.kraiem@fphm.u-monastir.tn.*

[b] *Inonu University, Faculty of Arts and Sciences, Department of Chemistry, Malatya, 44280, Turkey.*

[c] *Research Laboratory of Environmental Sciences and Technologies (LR16ES09), Higher Institute of Environmental Sciences and Technology, University of Carthage, Hammam-Lif, Tunisia.*

1. Chemistry

1.1. General data

All chemicals and solvents obtained from commercial suppliers were used without further purification. Analytical thin layer chromatography (TLC) was performed on Merck 60F-254 precoated silica gel sheets (0.25 mm). The plates were visualized by irradiation with UV light, followed by spraying with solution of KI. For column chromatography, 60–120 mesh silica gel and/or Celite 545 were used. Melting points were measured with Kofler heating bench and are uncorrected.^{1}H NMR,^{13}C and ^{19}F NMR spectra were recorded on a Bruker AV 500 MHz, AV 400 MHz or AV 300 MHz, using $CDCl_3$ or DMSO-d_6 as solvent. Chemical shifts (δ) are expressed in ppm relative to residual solvents ($CDCl_3$: δ_H = 7.26, δ_C =77.16; DMSO-d_6: δ_H = 2.50, δ_C = 39.52). In all ^{1}H NMR spectra, the number of protons for each signal and coupling constants values (Hertz) are indicated. Multiplicity is also reported and designated by the following abbreviations: s (singlet), d (doublet), (dd) doublet of doublets, t (triplet), q (quartet) m (multiplet).

1.2. General procedure

1.2.1. Synthesis of *N*-sulfonylimines 1a-j

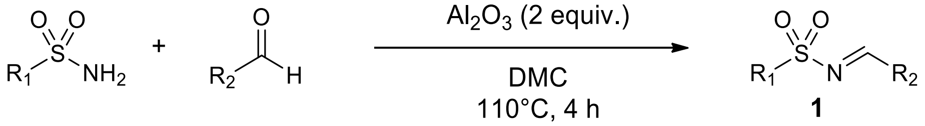

N-sulfonylimines were prepared according to the method developed by our group[1].A mixture of sulfonamide (1.2 mmol), aldehyde (1 mmol), and aluminum oxide (2 equiv.) in 1 mL of anhydrous DMC was stirred and heated in an 8 mL pressure tube at 110 °C for 4 hours. The progress of the reaction was monitored by TLC (cyclohexane/AcOEt (7:3)). After the reaction was complete, the crude mixture was filtered, and Al_2O_3 was thoroughly washed with 4 mL of hot DMC (60 °C). The collected filtrate was concentrated under reduced pressure and crystallized from ethanol/water to afford pure *N*-sulfonylimines **1**.

1.2.2. Synthesis of *N*-sulfonyloxaziridines 2a-j

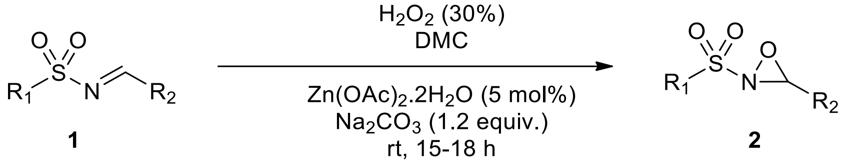

N-sulfonyloxaziridines were prepared according to the method developed by our group[2]. To a solution of *N*-sulfonylimine **1** (0.5 mmol) in DMC (1.5 mL) was added $Zn(OAc)_2.2H_2O$ (5mol%) and Na_2CO_3 (1.2 equiv). Then a 10 equiv. of 30 % hydrogen peroxide solution was added over a period of 5 min. The mixture was stirred at room temperature until disappearance of the imine (15-18h). The reaction was monitored by TLC (cyclohexane: AcOEt (8:2)). The reaction mixture was then diluted with AcOEt, washed with a solution of sodium sulfite and extracted with AcOEt (3×2 mL). The organic phase was dried over $MgSO_4$ and concentrated on a rotary evaporator to obtain an oily residue that usually solidifies at rest. This residue was then filtered through a short pad of silica gel (cyclohexane: AcOEt) to yield pure product **2**.

1.2.3. Synthesis of *N*-acylsulfonamides 3a-j

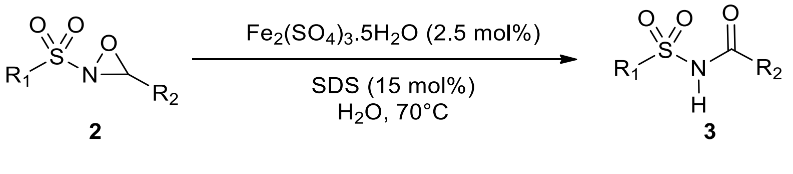

In a 8 mL sealed vial, $Fe_2(SO_4)_3.5H_2O$ (2.5 mol%) and SDS (15 mol%) was stirred in 1 mL of H_2O at room temperature for 5 min. Thereafter, *N*-sulfonyloxaziridine **2** (0.5 mmol) was added and the reaction was carried out at 70˚C until disappearance of the oxaziridine. The progress of the reaction was monitored by TLC (revealed with cyclohexane: AcOEt (7:3), and then sprayed with an aqueous solution of KI). After the completion of the reaction, 1mL of AcOEt was added and the mixture was centrifuged ((5min, 3000 rpm) ×3) to separate the organic and the

aqueous phases. Then, the residue was filtered through a short pad of silica gel and Celite. After evaporation of the solvent, the crude product was crystallized to yield the pure *N*-acylsulfonamides **3**.

N-acylsulfonamides **3a**[3],**3b**[3],**3c**[4], **3d**[5], **3e**[3], **3f**[6], **3h**[3], and **3j**[7]are known compounds and previously reported in the literature. *N*-acylsulfonamides, **3g** and **3i** are new compounds.

1.2.3.1. Synthesis of *N*-(phenylsulfonyl)benzamide (3a)

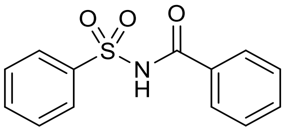

White solid (83%); crystallized from (ethanol/water); mp 147-149 °C; **^{1}H NMR:** δ_H (300 MHz; $CDCl_3$) 9.37 (1H, s), 8.17 (2H, d, *J* = 7.8 Hz), 7.82 (2H, d, *J* = 7.8 Hz), 7.65 (1H, t, *J* = 7.3 Hz), 7.55 (3H, t, *J* = 7.7 Hz), 7.41 (2H, t, *J* = 7.6 Hz); **^{13}C NMR:** δ_C (75 MHz; $CDCl_3$) 164.5, 138.8, 134.2, 133.6, 131.3, 129.1, 129.0, 128.7, 128.0.

1.2.3.2. *N*-tosylbenzamide (3b)

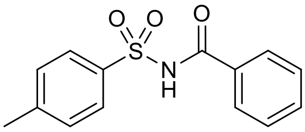

White solid (87%); crystallized from (ethanol/water); mp 144-146 °C; **^{1}H NMR:** δ_H (300 MHz; $CDCl_3$) 9.33 (1H, s), 8.05 (2H, d, *J* = 8.1 Hz), 7.82 (2H, d, *J* = 7.5 Hz), 7.54 (1H, t, *J* = 7.5 Hz), 7.41 (2H, t, *J* = 7.5 Hz), 7.34 (2H, d, *J* = 8.1 Hz), 2.43 (3H, s); **^{13}C NMR:** δ_C (75 MHz; $CDCl_3$) 164.5, 145.3, 135.8, 133.5, 131.4, 129.7, 129.0, 128.8, 128.0, 21.8.

1.2.3.3. *N*-((4-fluorophenyl)sulfonyl)benzamide (3c)

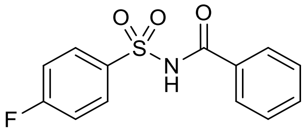

White solid (96%); crystallized from (ethanol/water); mp 176-178°C; **^{1}H NMR:** δ_H (300 MHz; $CDCl_3$) 9.10 (1H, s), 8.19 (2H, dd, *J* = 8.6 Hz, *J* = 5.0 Hz), 7.80 (2H, d, *J* = 7.5 Hz), 7.57 (1H, t, *J* = 7.4 Hz), 7.44 (2H, t, *J* = 7.5 Hz), 7.23 (2H, t, *J* = 8.6 Hz); **^{13}C NMR:** δ_C (75 MHz; $CDCl_3$) 166.2 (d, *J*

= 255.0), 164.4, 134.7 (d, *J* = 3.0 Hz), 133.8, 131.9 (d, *J* = 9.8 Hz), 131.3, 129.2, 128.0, 116.4 (d, *J* = 22.9 Hz); **^{19}F NMR:** δ_F (376 MHz, CDCl3,) -102.48.

1.2.3.4. 4-fluoro-*N*-(phenylsulfonyl)benzamide (3d)

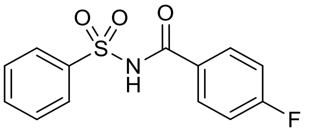

White solid (81%);crystallized from (ethanol/water); mp 158-160 °C; **^{1}H NMR:** δ_H (300 MHz; $CDCl_3$) 9.38 (1H, s), 8.16 (2H, d, *J* = 7.8 Hz), 7.86 (2H, dd, *J* = 8.4 Hz, *J* = 5.4 Hz), 7.66 (2H, t, *J* = 7.4 Hz), 7.56 (2H, t, *J* = 7.6 Hz), 7.09 (2H, t, *J* = 8.4 Hz); **^{13}C NMR:** δ_c (75 MHz; $CDCl_3$) 166.1 (d, *J* = 254.3 Hz), 163.5, 138.7, 134.3, 130.7 (d, *J* = 9.0 Hz), 129.2, 128.7, 127.6 (d, *J* = 3.0 Hz), 116.3 (d, *J* = 22.5 Hz); **^{19}F NMR:** δ_F (376 MHz, $CDCl_3$) -103.89.

1.2.3.5. 4-fluoro-*N*-tosylbenzamide (3e)

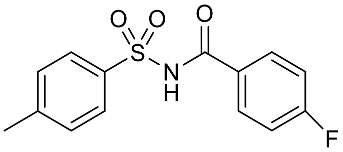

White solid(96%); crystallized from (ethanol/water); mp 160-162°C; **^{1}H NMR:** δ_H (500 MHz; $CDCl_3$) 9.45 (1H, s), 8.04 (2H, d, *J* = 8.4 Hz), 7.87 (2H, dd, *J* = 9.0 Hz, *J* = 5.0 Hz), 7.36 (2H, d, *J* = 8.0 Hz) 7.09 (t, 2H, *J* = 8.5 Hz), 2.44 (3H, s); **^{13}C NMR:** δ_c (125 MHz; $CDCl_3$) 166.0 (d, *J* =254.3 Hz), 163.5, 145.6, 135.4, 130.7 (d, *J* = 9.5 Hz), 129.8, 128.8, 127.4 (d, *J* = 3.0 Hz), 116.2 (d, *J* = 22.0 Hz), 21.9; **^{19}F NMR:** δ_F (376 MHz, $CDCl_3$) -104.08.

1.2.3.6. *N*-tosyl-4-(trifluoromethyl)benzamide (3f)

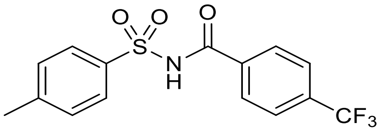

White solid (80%); crystallized from (ethanol/water); mp 187-189 °C; **^{1}H NMR:** δ_H (300 MHz; $CDCl_3$) 9.48 (1H, s), 8.04 (2H, d, *J* = 8.1 Hz), 7.95 (2H, d, *J* = 8.1 Hz), 7.68 (2H, d, *J* = 8.1 Hz), 7.36 (2H, d, *J* = 8.1 Hz), 2.45 (3H, s); **^{13}C NMR:** δ_c (75 MHz; $CDCl_3$) 163.5, 145.8, 135.4 (q, *J* = 33.0 Hz), 134.7, 129.9, 128.9, 128.8, 128.6, 126.1(q, *J* = 3.7 Hz), 123.5 (q, *J* = 271.1 Hz), 21.8; **^{19}F NMR:** δ_F (282 MHz, $CDCl_3$)-63.35.

1.2.3.7. *N*-((4-fluorophenyl)sulfonyl)-4-(trifluoromethyl)benzamide (3g)

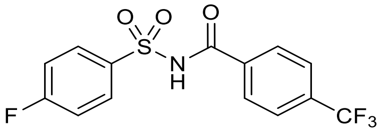

White solid (98%); crystallized from (ethanol/diethyl ether); mp148-150 °C; **^{1}H NMR:** δ_H (400 MHz; $CDCl_3$); 9.44 (1H, s), 8.22 (2H, dd, *J* = 8.8 Hz, *J* = 4.8 Hz),7.96 (2H, d, *J* = 8.0 Hz), 7.73 (2H, d, *J* = 8.4 Hz), 7.28 (2H, t, *J* = 8.5 Hz); **^{13}C NMR:**δ_c (100 MHz; $CDCl_3$) 166.2 (d, *J* = 256.0 Hz),163.4, 135.3 (q, *J* = 32.8 Hz), 134.3, 134.0 (d, *J* = 3.2 Hz), 131.9 (d, *J* = 9.8 Hz), 128.5, 126.2 (q, *J* = 3.6 Hz), 123.4 (q, *J* = 271.0), 116.6 (d, *J* = 22.7 Hz); **^{19}F NMR:** δ_F (376 MHz, $CDCl_3$) -63.30, -101.70; HRMS (+ESI) calculated for $C_{14}H_9F_4NO_3S$ $[M+H]^+$: 348.0317, found: 348.0315.

1.2.3.8. 4-fluoro-*N*-(methylsulfonyl)benzamide (3h)

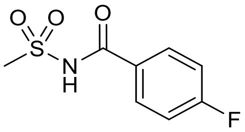

White solid(86%); crystallized from (ethanol/water); mp 188-190 °C; **^{1}H NMR:**δ_H (400 MHz; DMSO) 12.17 (1H, s), 8.03 (2H, dd, *J*= 8.8 Hz, *J*= 5.6Hz), 7.36 (2H, t, *J*= 8.8 Hz), 3.38 (3H, s); **^{13}C RMN:** δ_c (100 MHz; DMSO);165.3, 165.0 (d, *J* = 250.0 Hz), 131.4 (d, *J* = 9.4 Hz), 128.2 (d, *J* = 3.0 Hz), 115.7 (d, *J* = 22.0 Hz), 41.3; **^{19}F NMR:** δ_F (376 MHz, DMSO) -105.98.

1.2.3.9. 3,4-difluoro-*N*-(methylsulfonyl)benzamide (3i)

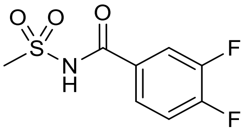

White solid (98%); crystallized from (ethanol/water); mp 143-145 °C; **^{1}H NMR:** δ_H (400 MHz; DMSO) 12.24 (1H, s), 8.10-7.96 (1H, m),7.92-7.80 (1H, m), 7.69-7.55 (1H,m), 3.38 (3H, s); **^{13}C NMR:** δ_C (100 MHz; DMSO)164.3 (d, *J* = 1.0 Hz), 152.5 (dd, *J* = 252.0 Hz, *J* = 12.0 Hz), 149.1 (dd, *J*= 246.0 Hz, *J* = 13.0 Hz), 129.1 (t, *J* = 4.0 Hz), 126.3 (dd, *J* = 8.0 Hz, *J* = 4.0 Hz), 118.0 (d, *J* = 19.0 Hz), 117.9 (d, *J* = 18.0 Hz), 41.3; **^{19}H NMR:** δ_F(376 MHz, DMSO)-131.32 (d, *J* = 22.2 Hz), -137.20 (d, *J* = 22.3 Hz); HRMS (+ESI) calculated for C8H7F2NO3S [M+H]+ : 236.0192, found: 236.0185.

1.2.3.10. *N*-(methylsulfonyl)-4-(trifluoromethyl)benzamide (3j)

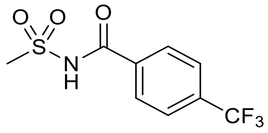

White solid (94%); crystallized from (ethanol/diethyl ether); mp 148-150 °C; **^{1}H NMR:**δ_H (400 MHz; DMSO) 12.43 (1H, s); 8.13 (2H, d, *J*=8.0 Hz), 7.91 (2H, d, *J* = 8.0 Hz) 3.40 (3H, s); **^{19}F NMR:** δ_F (376 MHz) -61.62.

2. Pharmacological evaluation: *In vivo* anti-inflammatory activity

2.1. Animals

Adult male Wistar rats (140-180 g) used in the anti-inflammatory screens were obtained from the Pasteur Institute (Tunis, Tunisia). They were housed at controlled temperature (22 ± 2°C) with free access to standard food pellets and water. Twelve hours before the experiments, the rats were fasted and allowed access to water *ad libitum*. All animal experiments were carried out in accordance with the European Union Regulations (Directive 86/609 CEE).

2.2. Method

Effects of *N*-acylsulfonamides **3a-j** on carrageena*N*-induced paw edema in rats were evaluated according to the method developed by Winter et *al*[8]. Animals were divided into twelve groups of 6 animals each. The control group received normal saline (2.5 mL/kg, *i.p*), the standard group received Diclofenac (25 mg/kg, *i.p*) and the test groups received the synthesized compounds **3a-j** (50 mg/kg, *i.p*). 30 minutes later, carrageenan suspension (1% w/v, in 0.9% saline solution) was subcutaneously injected into the left hind paw. The volume of paw edema was measured by plethysmometer (Ugo Basile 7150, Italy) before (V_0) and after injection of carrageenan at 1, 2, 3, 4 and 5 h (V_T). The percentage of paw edema inhibition was calculated using the following formula:

$$\%\,inhibition = \frac{(\mathrm{VT}-\mathrm{V0})\ \mathrm{control} - \ (\mathrm{VT}-\mathrm{V0})\ \mathrm{treated\ group}}{(\mathrm{VT}-\mathrm{V0})\ \mathrm{control}} \times 100$$

3. ADMET profile

The evaluation of pharmacokinetics and toxicity proprieties of *N*-acylsulfonamide derivatives were assessed using web-based services ADMET-AI that relies on Chemprop-RDKit graph

neural network (GNN) architecture (https://admet.ai.greenstonebio.com/) and ProTox 3.0 (https://tox.charite.de/protox3/)that relies on molecular similarities.

References

1 Z. Litim, H. Slimi, T. Ollevier and J. Kraïem, *RSC Adv.*, 2023, **13**, 4431–4435.
2 J. Kraïem, D. Ghedira and T. Ollevier, *Green Chem.*, 2016, **18**, 4859–4864.
3 W. Yang, D. Huang, X. Zeng, J. Zhang, X. Wang and Y. Hu, *Tetrahedron*, 2019, **75**, 381–386.
4 Y. Wang, K. Sarris, D. R. Sauer and S. W. Djuric, *Tetrahedron Lett.*, 2007, **48**, 5181–5184.
5 P. A. Suchetan, S. Sreenivasa, K. S. Srivishnu, H. N. Lakshmikantha, G. M. Supriya, S. Naveen and N. K. Lokanath, *Z. Für Krist. - Cryst. Mater.*, 2015, **230**, 543–550.
6 T. M. U. Ton, C. Tejo, S. Tania, J. W. W. Chang and P. W. H. Chan, *J. Org. Chem.*, 2011, **76**, 4894–4904.
7 M. Frings, C. Bolm, A. Blum and C. Gnamm, *Eur. J. Med. Chem.*, 2017, **126**, 225–245.
8 C. A. Winter, E. A. Risley and G. W. Nuss, *Proc. Soc. Exp. Biol. Med.*, 1962, **111**, 544–547.

^{1}H NMR, ^{13}C NMR and ^{19}F NMR spectra of compounds 3a-j

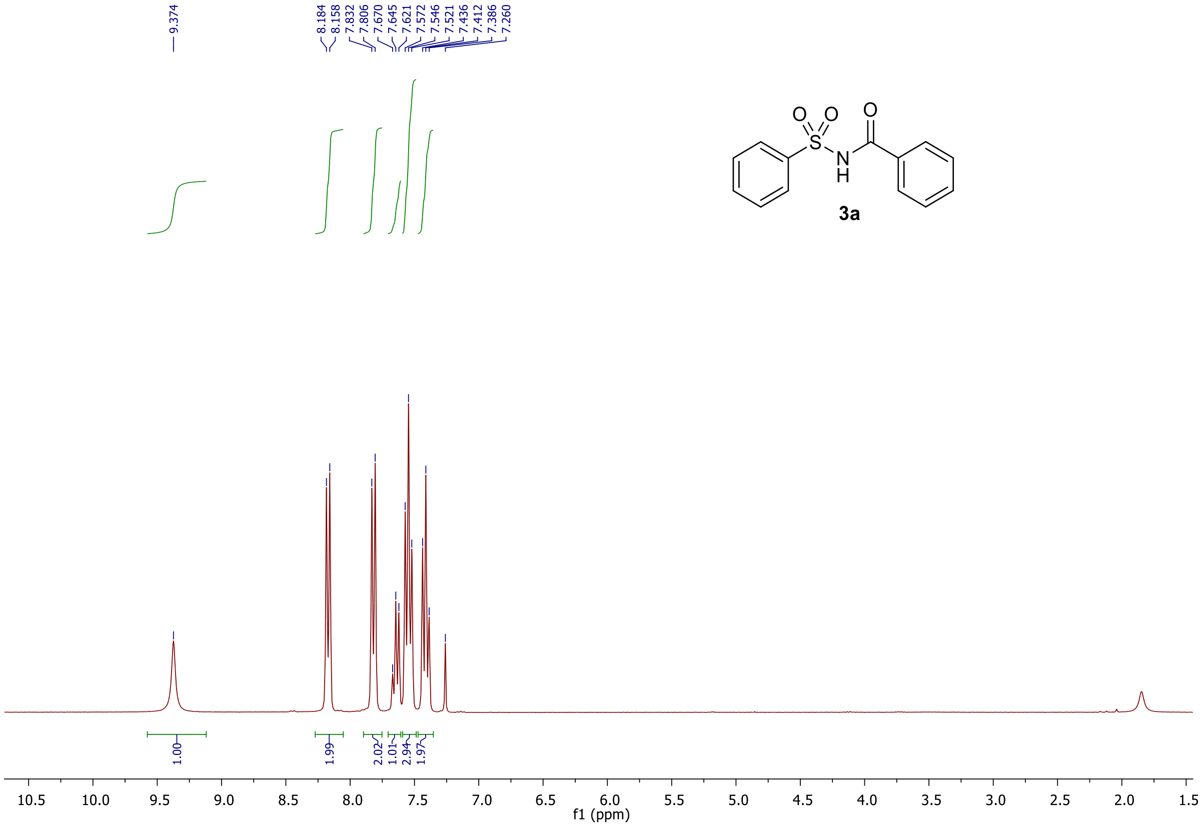

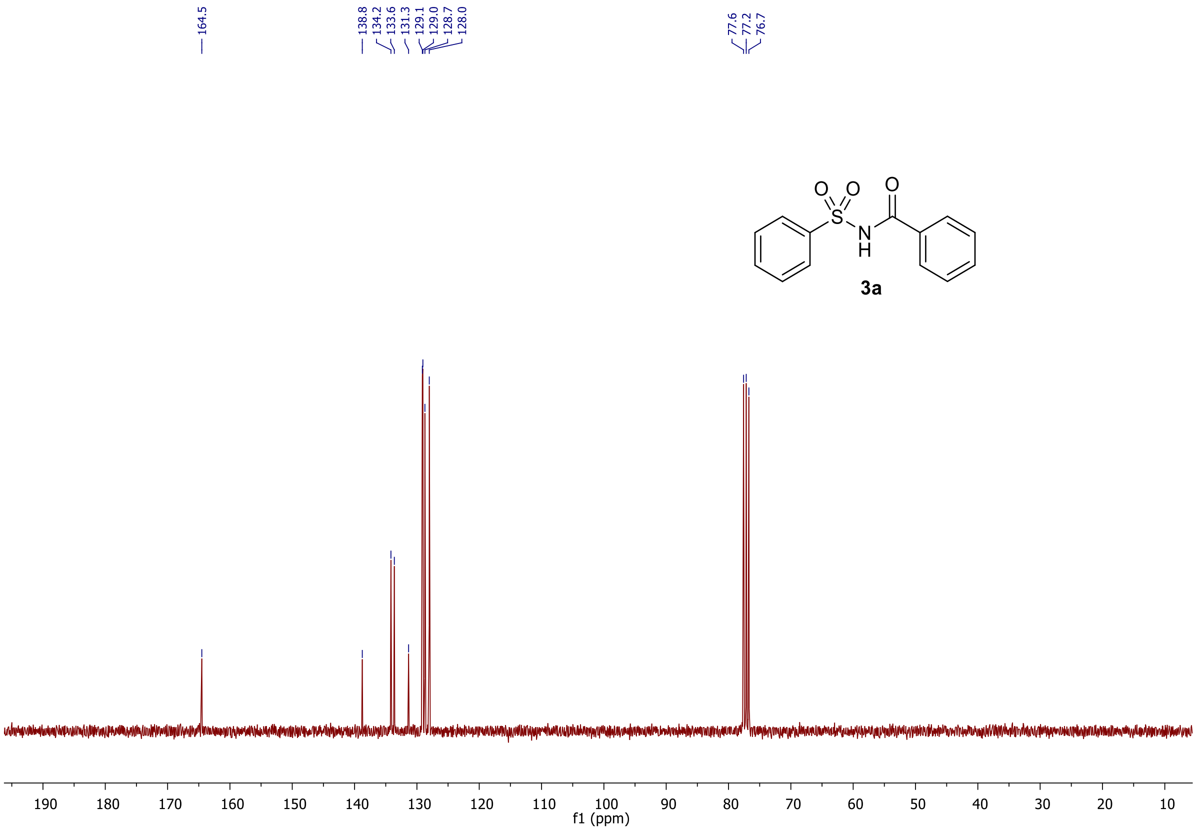
164.5
138.8
134.2
133.6
131.3
129.1
129.0
128.7
128.0
77.6
77.2
76.7
3a
190 180 170 160 150 140 130 120 110 100 90 80 70 60 50 40 30 20 10
f1 (ppm)

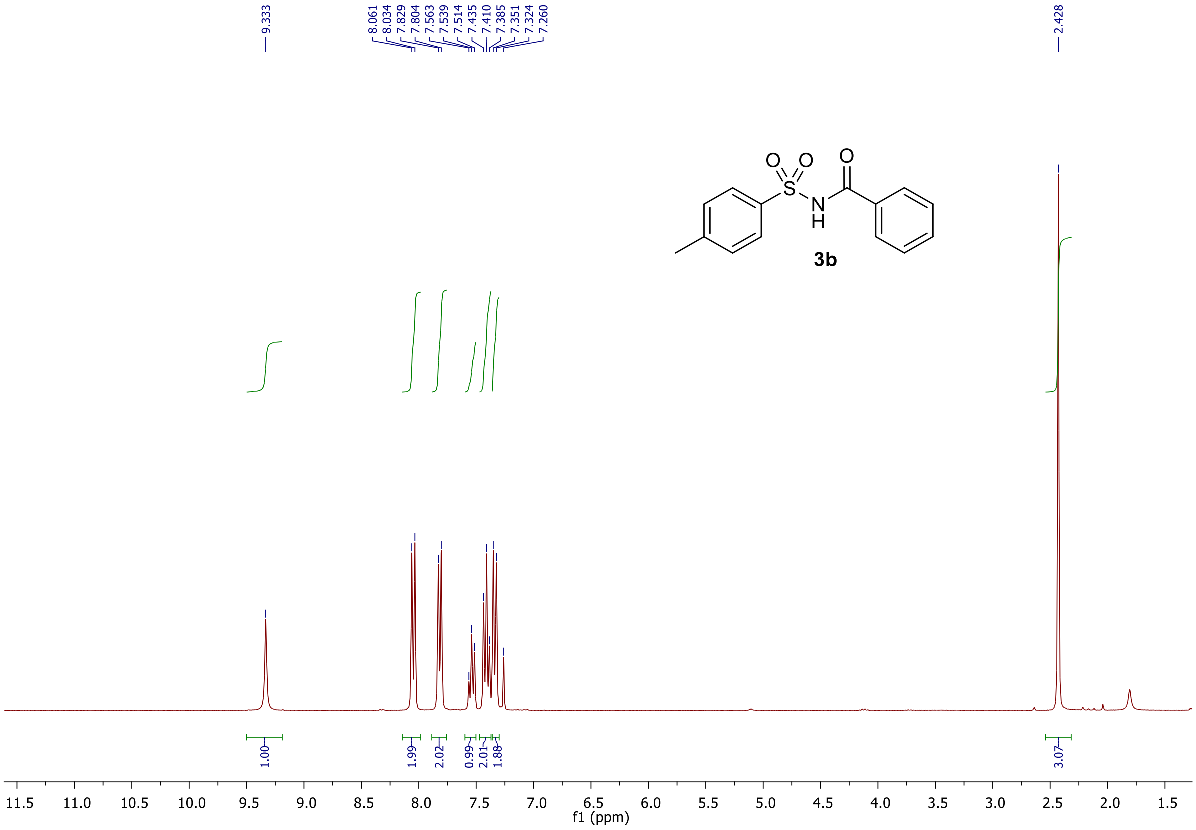
9.333
8.061
8.034
7.829
7.804
7.563
7.539
7.514
7.435
7.410
7.385
7.351
7.324
7.260
2.428
3b
1.00
1.99
2.02
0.99
2.01
1.88
3.07
11.5 11.0 10.5 10.0 9.5 9.0 8.5 8.0 7.5 7.0 6.5 6.0 5.5 5.0 4.5 4.0 3.5 3.0 2.5 2.0 1.5
f1 (ppm)

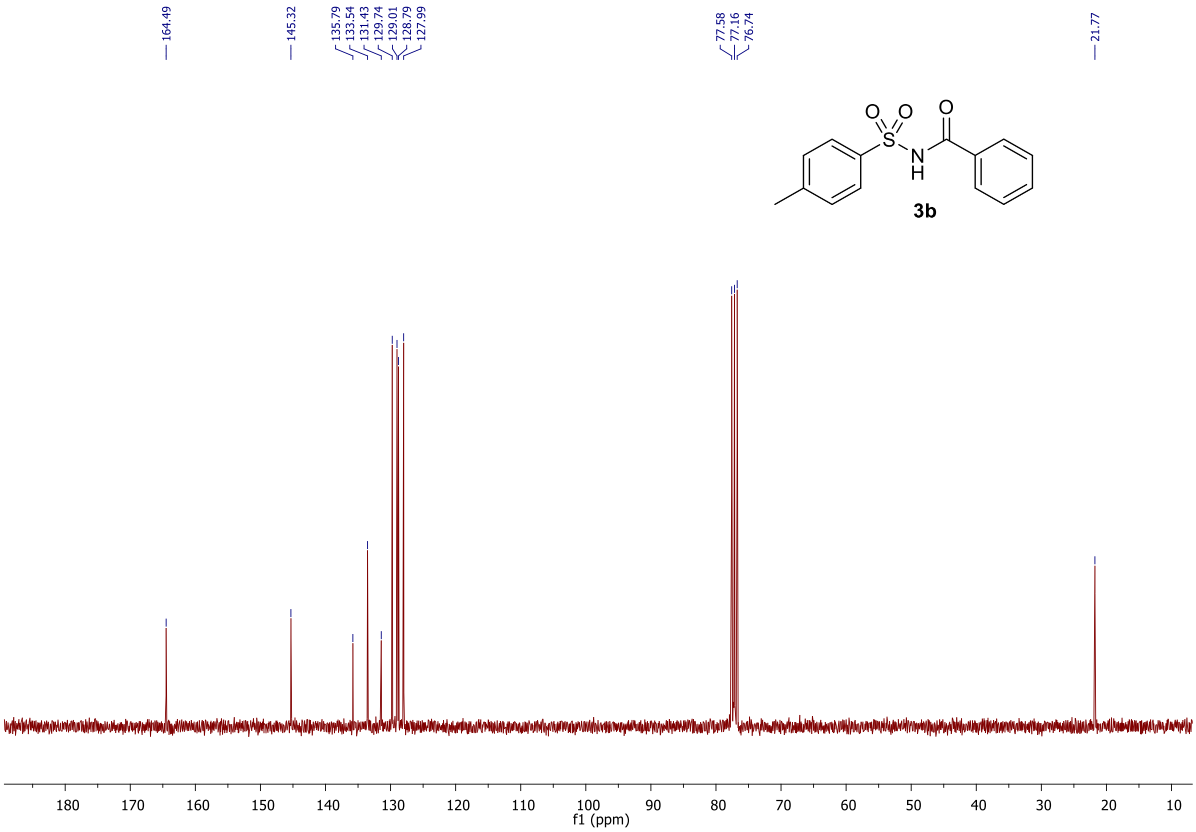

164.49
145.32
135.79
133.54
131.43
129.74
129.01
128.79
127.99
77.58
77.16
76.74
21.77
3b
f1 (ppm)

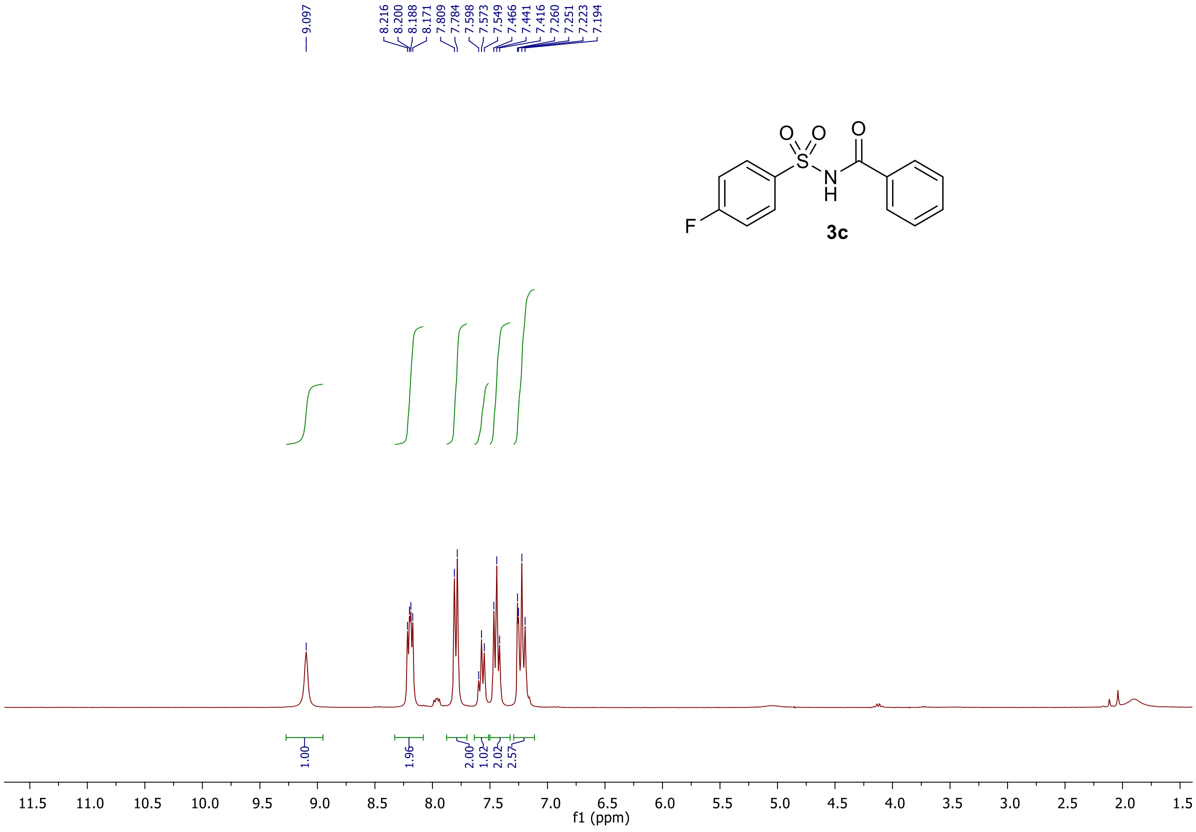

9.097
8.216
8.200
8.188
8.171
7.809
7.784
7.598
7.573
7.549
7.466
7.441
7.416
7.260
7.251
7.223
7.194
3c
1.00
1.96
2.00
1.02
2.02
2.57
f1 (ppm)

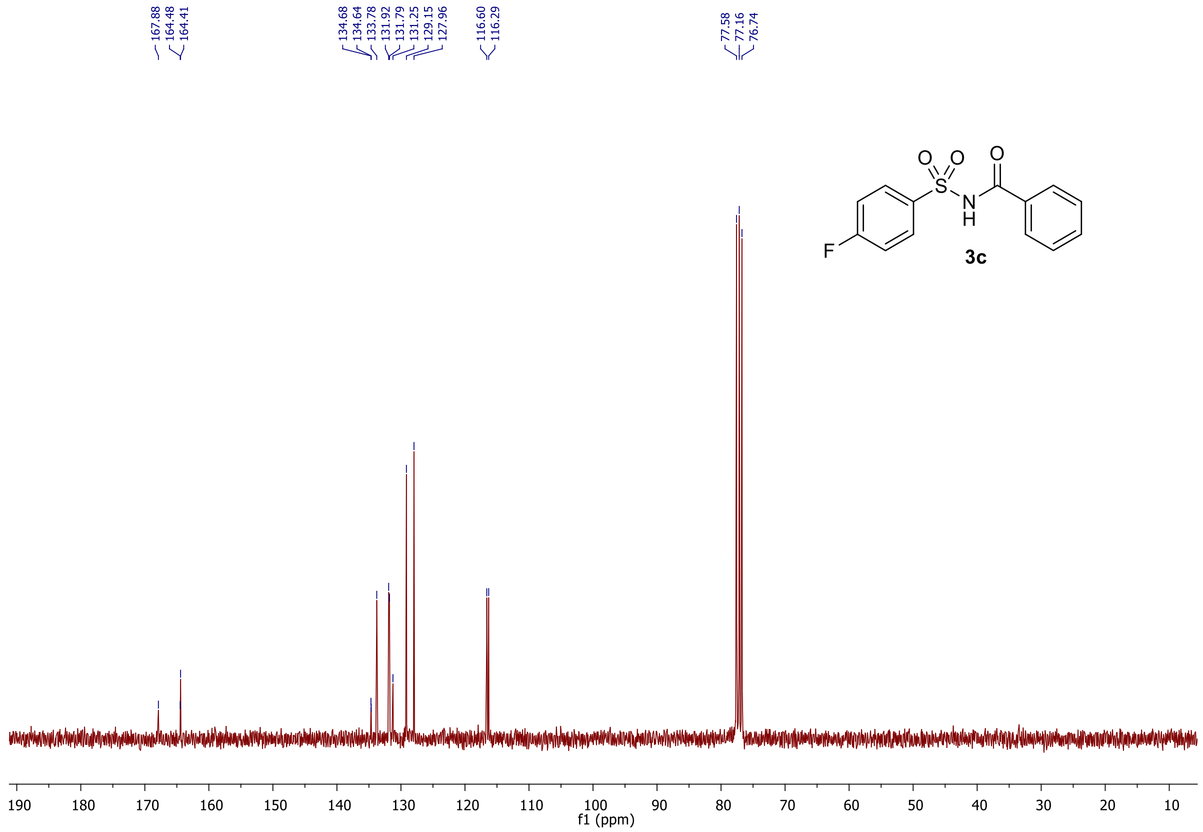
167.88
164.48
164.41
134.68
134.64
133.78
131.92
131.79
131.25
129.15
127.96
116.60
116.29
77.58
77.16
76.74
3c
190 180 170 160 150 140 130 120 110 100 90 80 70 60 50 40 30 20 10
f1 (ppm)

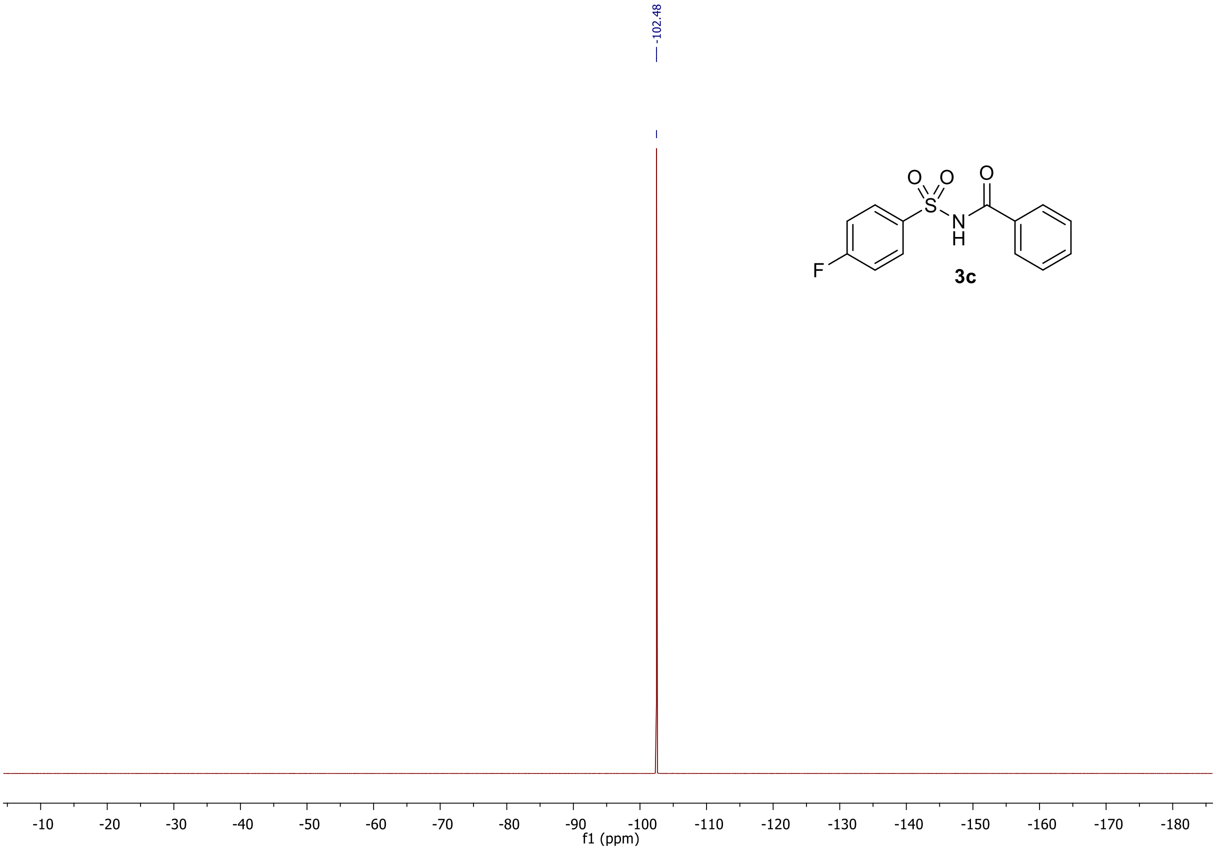
-102.48
3c
-10 -20 -30 -40 -50 -60 -70 -80 -90 -100 -110 -120 -130 -140 -150 -160 -170 -180
f1 (ppm)

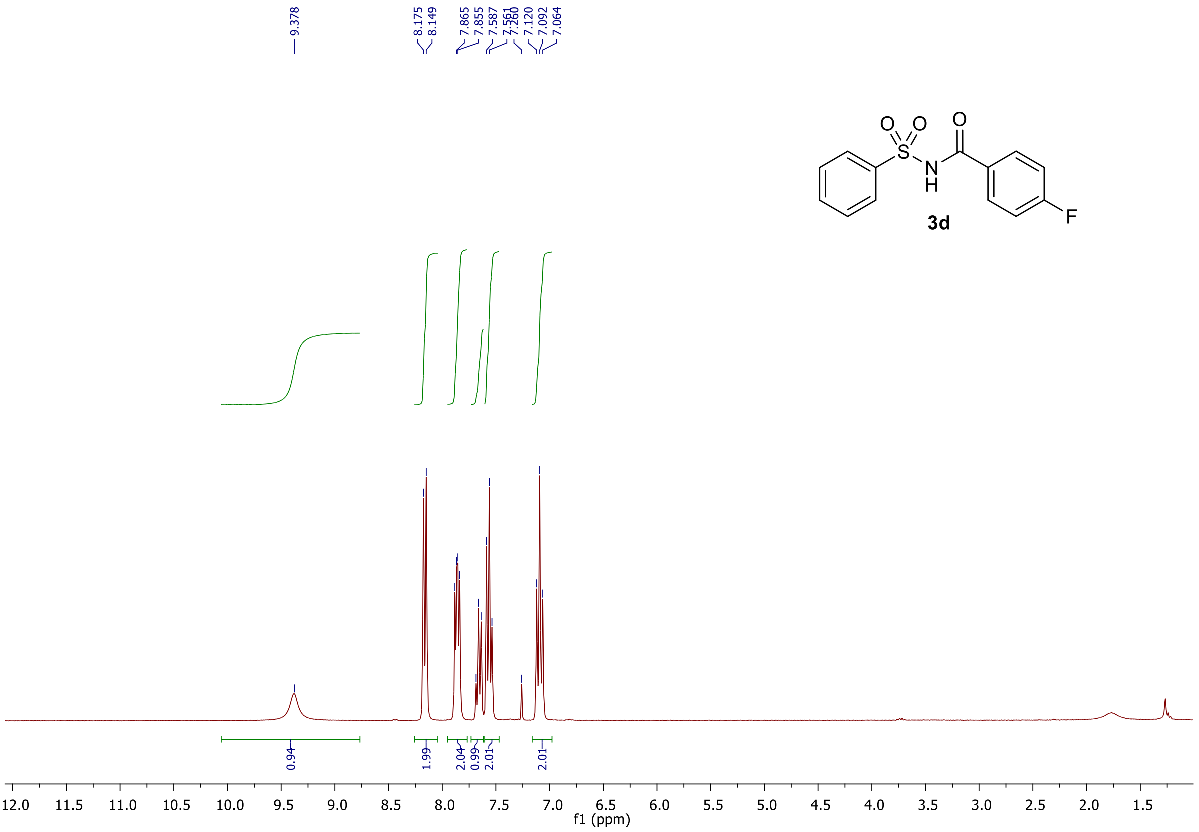
9.378
8.175
8.149
7.865
7.855
7.587
7.561
7.260
7.120
7.092
7.064
3d
F
0.94
1.99
2.04
0.99
2.01
2.01
f1 (ppm)

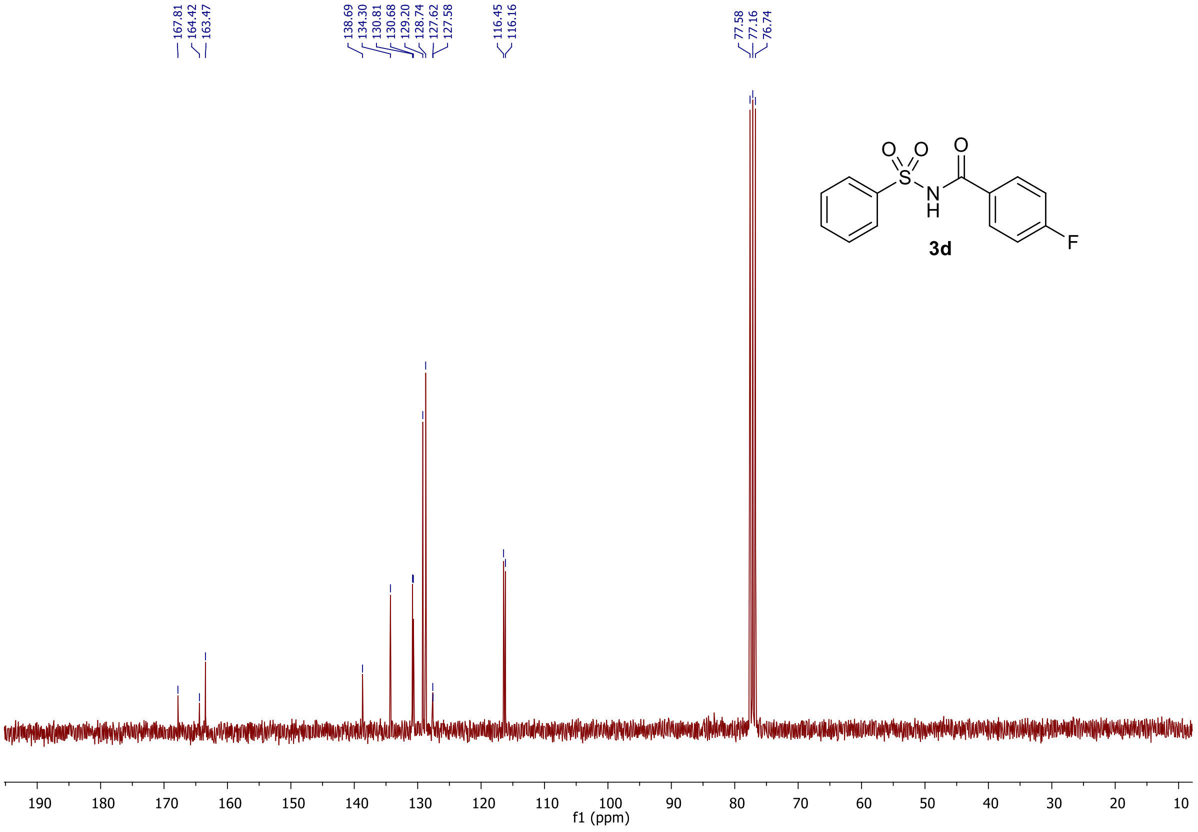
167.81
164.42
163.47
138.69
134.30
130.81
130.68
129.20
128.74
127.62
127.58
116.45
116.16
77.58
77.16
76.74
3d
F
f1 (ppm)

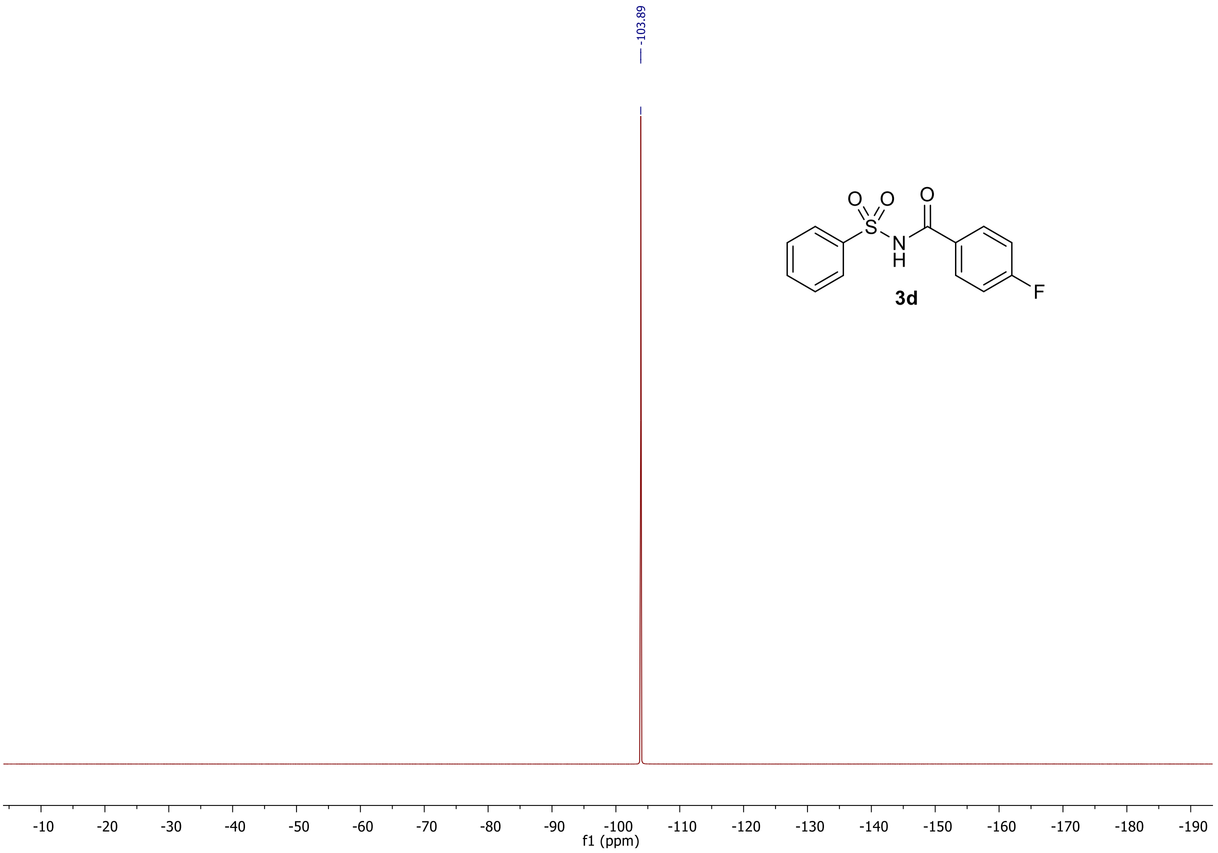

-103.89
O O O
S N H
F
-10 -20 -30 -40 -50 -60 -70 -80 -90 -100 -110 -120 -130 -140 -150 -160 -170 -180 -190
f1 (ppm)

3d

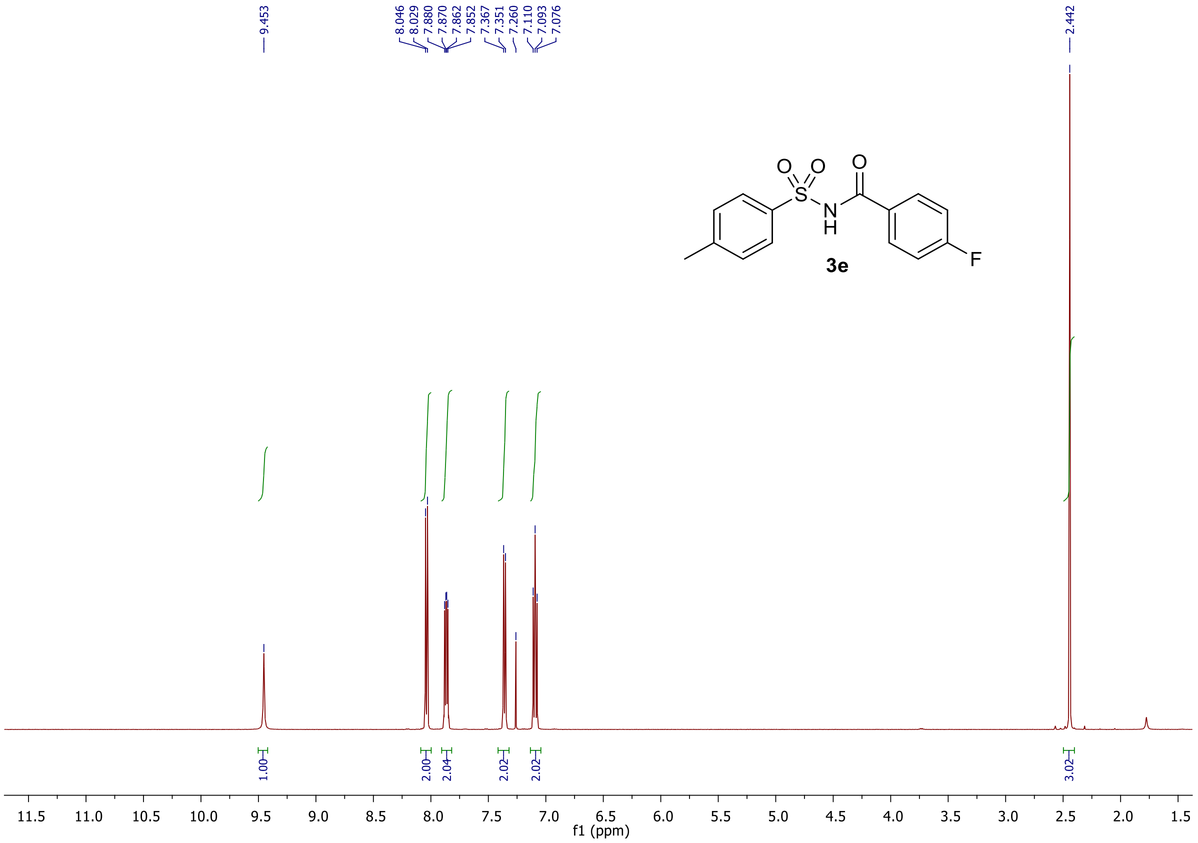

9.453
8.046 8.029 7.880 7.870 7.862 7.852 7.367 7.351 7.260 7.110 7.093 7.076
2.442
O O O
S N H
F
1.00 2.00 2.04 2.02 2.02 3.02
11.5 11.0 10.5 10.0 9.5 9.0 8.5 8.0 7.5 7.0 6.5 6.0 5.5 5.0 4.5 4.0 3.5 3.0 2.5 2.0 1.5
f1 (ppm)

3e

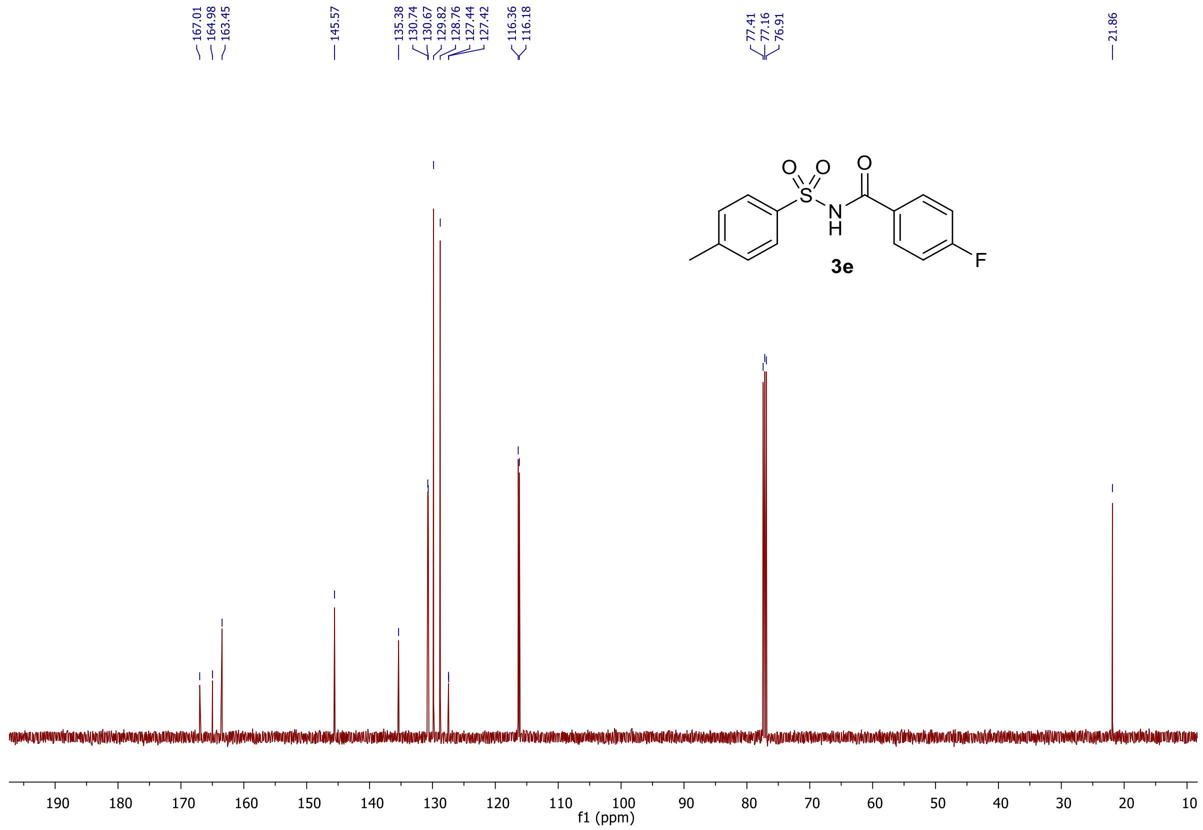

167.01
164.98
163.45
145.57
135.38
130.74
130.67
129.82
128.76
127.44
127.42
116.36
116.18
77.41
77.16
76.91
21.86
3e
f1 (ppm)

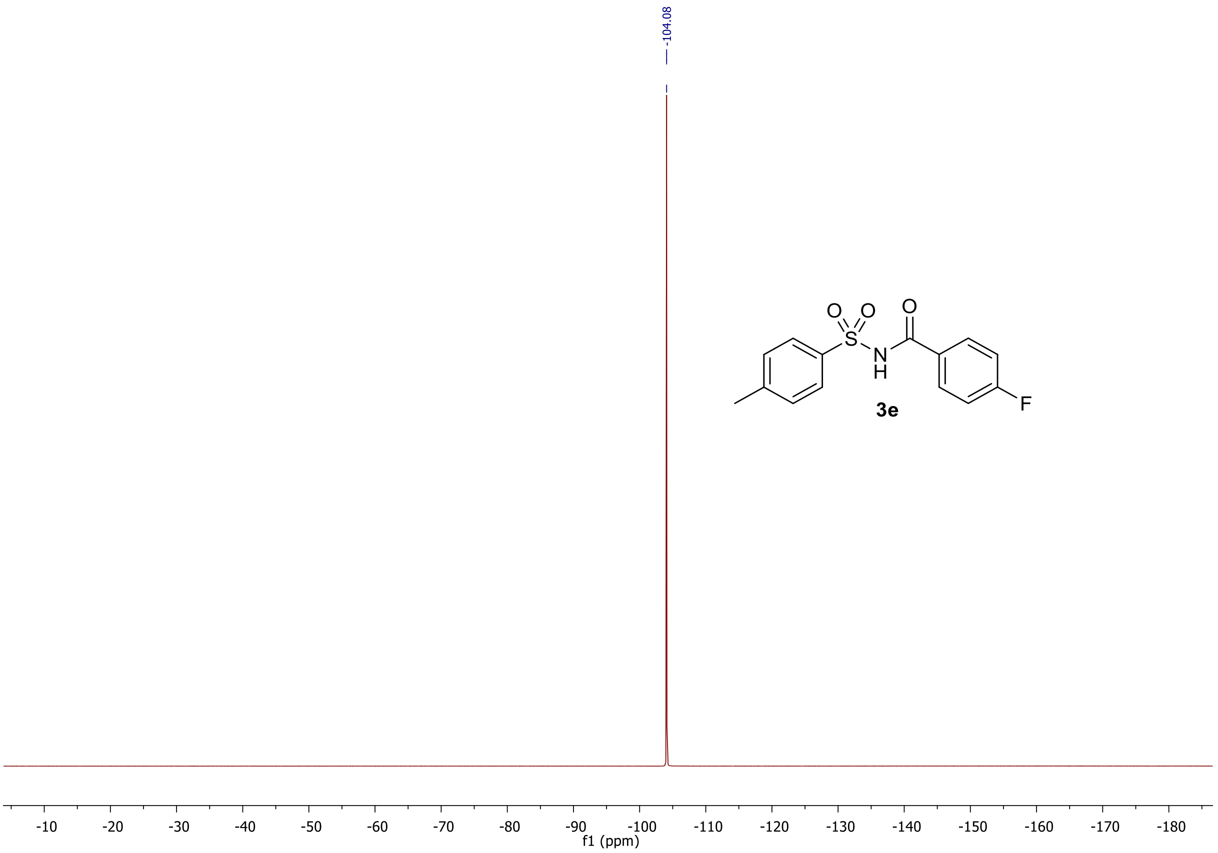

-104.08
3e
f1 (ppm)

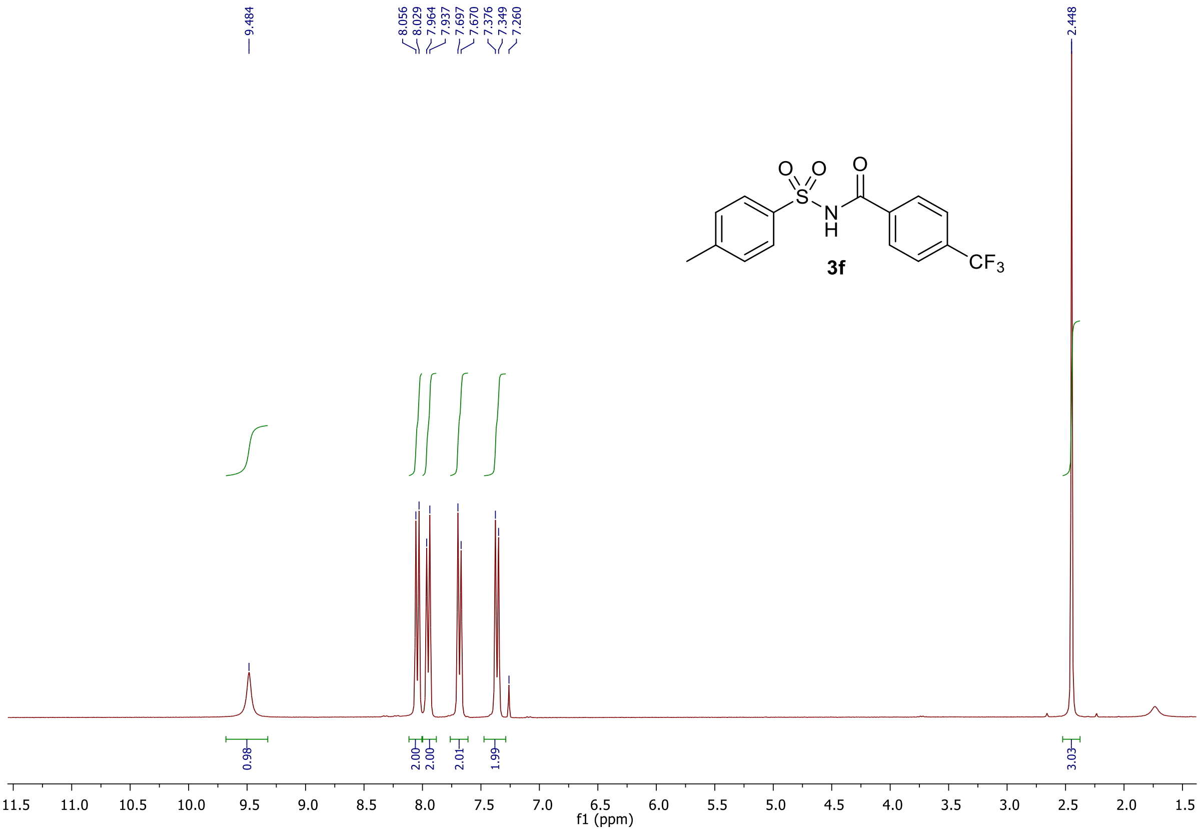
9.484
8.056
8.029
7.964
7.937
7.697
7.670
7.376
7.349
7.260
2.448
3f
CF3
0.98
2.00
2.00
2.01
1.99
3.03
f1 (ppm)

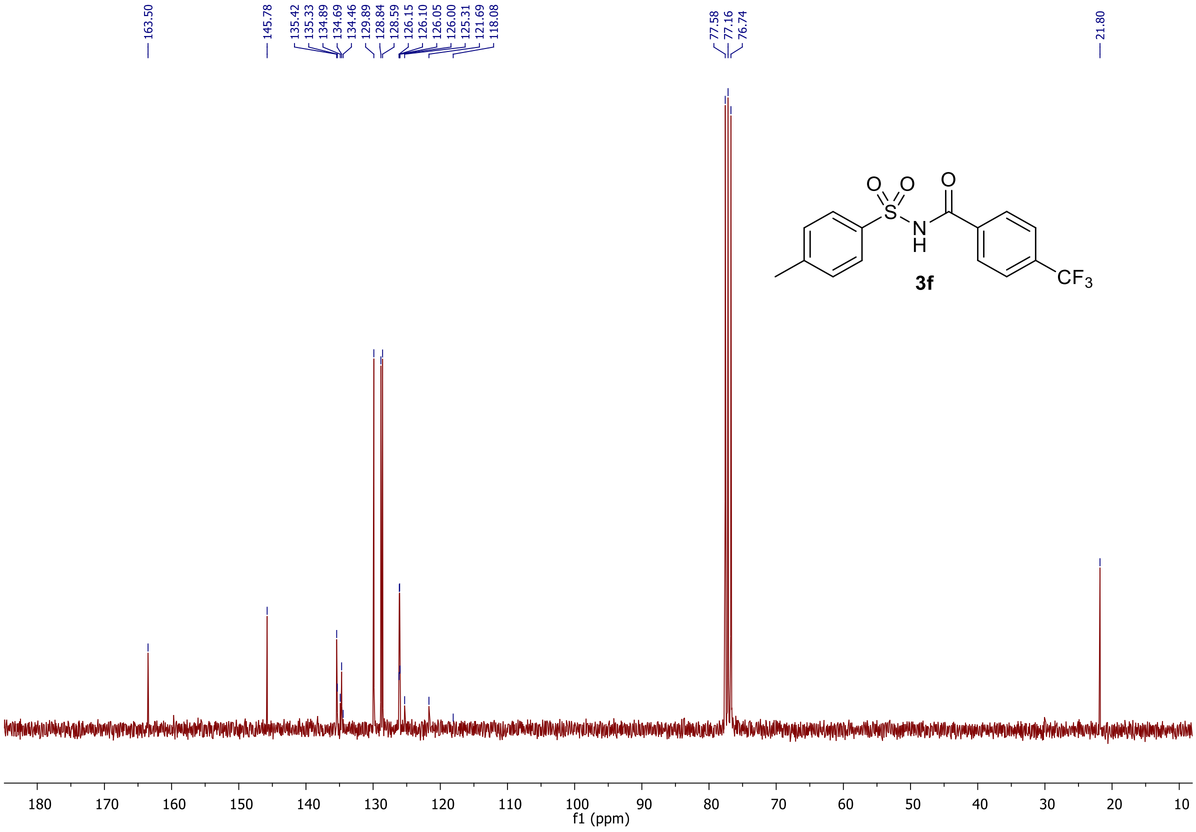
163.50
145.78
135.42
135.33
134.89
134.69
134.46
129.89
128.84
128.59
126.15
126.10
126.05
126.00
125.31
121.69
118.08
77.58
77.16
76.74
21.80
3f
CF3
f1 (ppm)

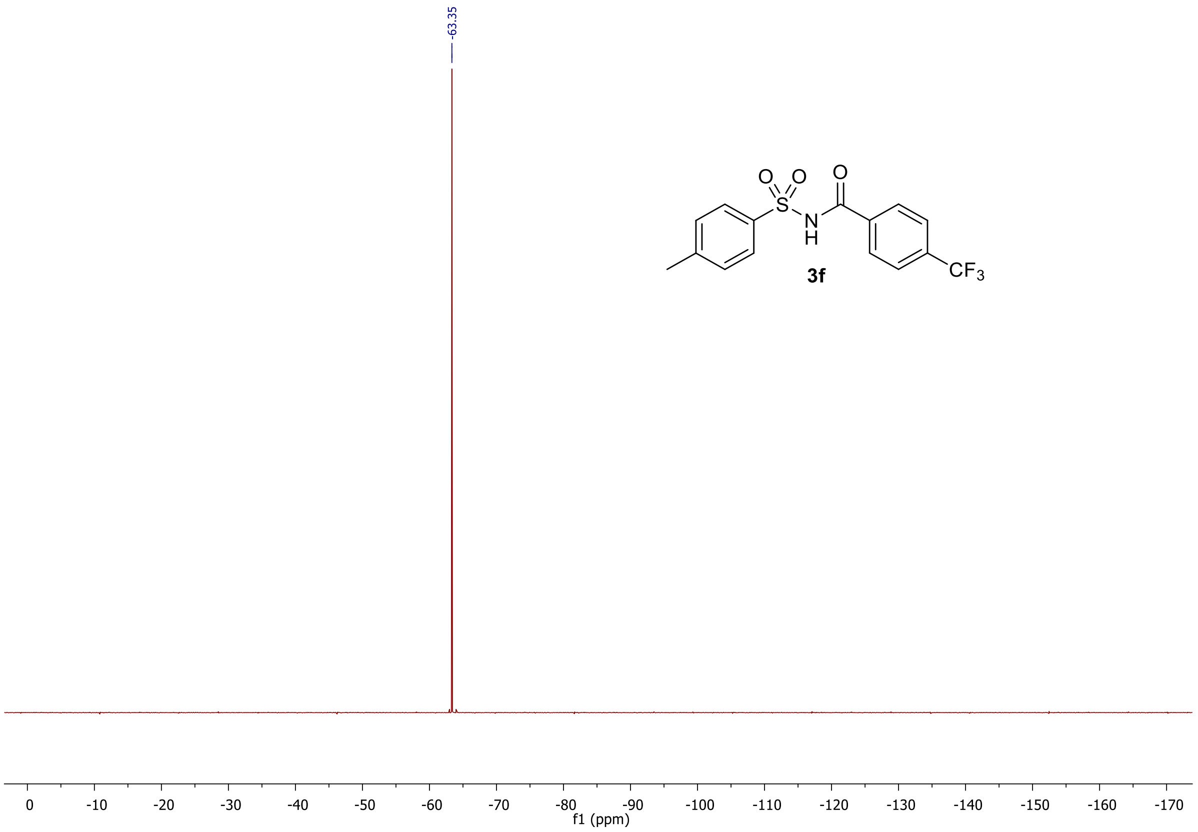
-63.35
3f
CF3
f1 (ppm)

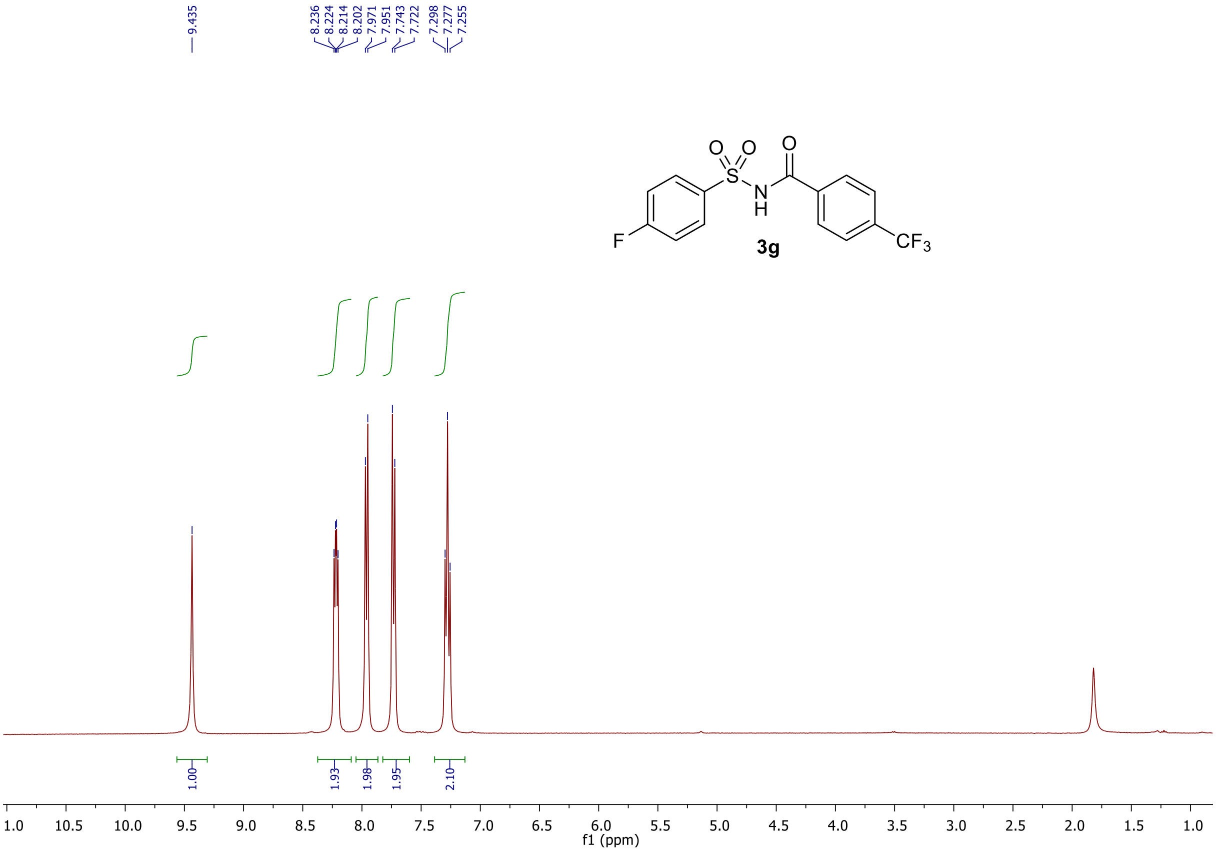
9.435
8.236
8.224
8.214
8.202
7.971
7.951
7.743
7.722
7.298
7.277
7.255
3g
F
CF3
1.00
1.93
1.98
1.95
2.10
f1 (ppm)

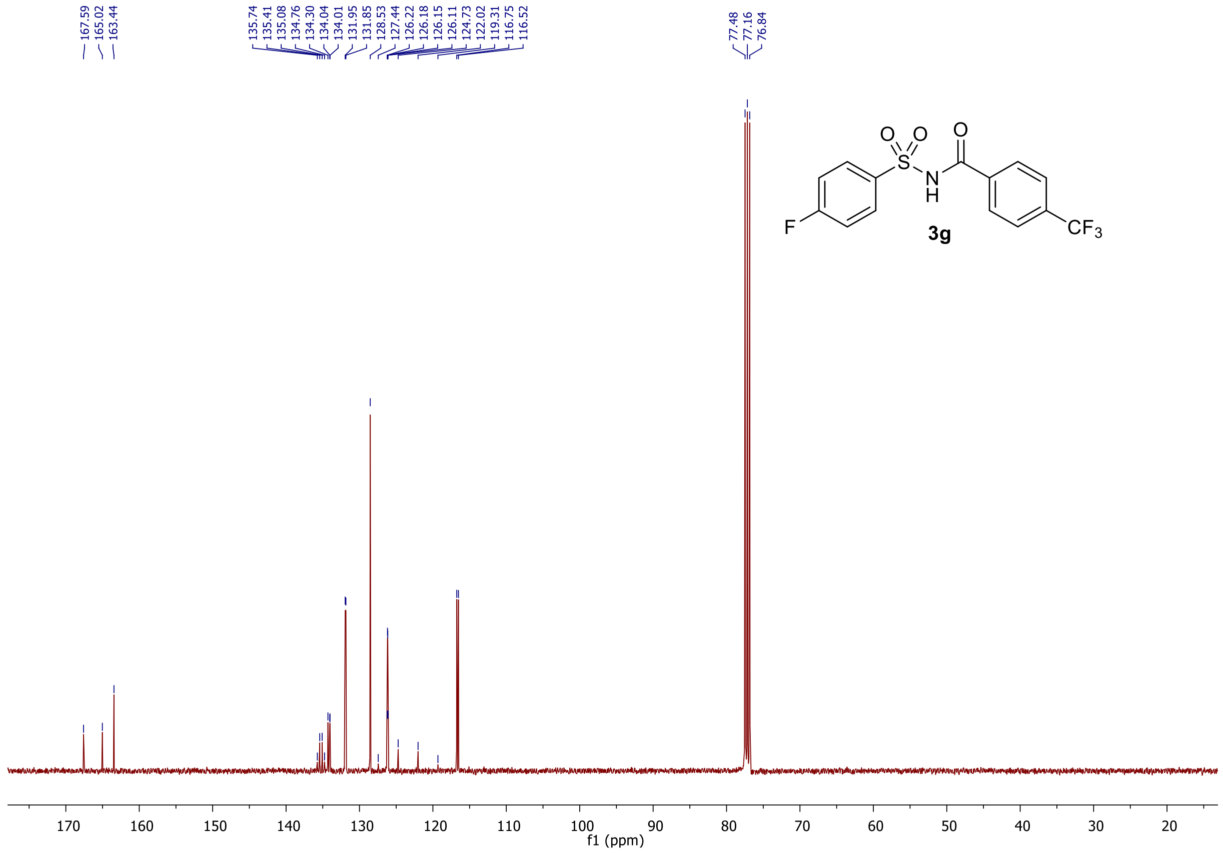
167.59
165.02
163.44
135.74
135.41
135.08
134.76
134.30
134.04
134.01
131.95
131.85
128.53
127.44
126.22
126.18
126.15
126.11
124.73
122.02
119.31
116.75
116.52
77.48
77.16
76.84
3g
f1 (ppm)

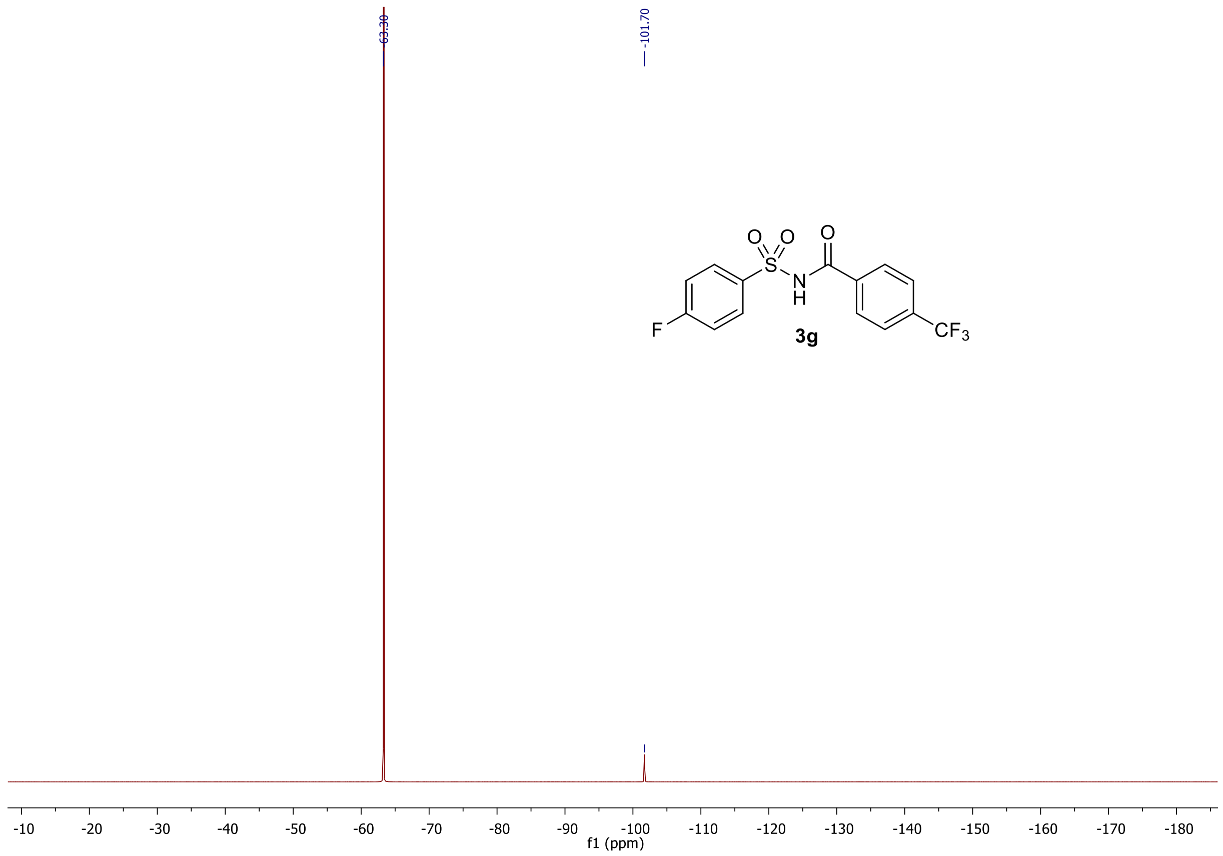
-63.30
-101.70
3g
f1 (ppm)

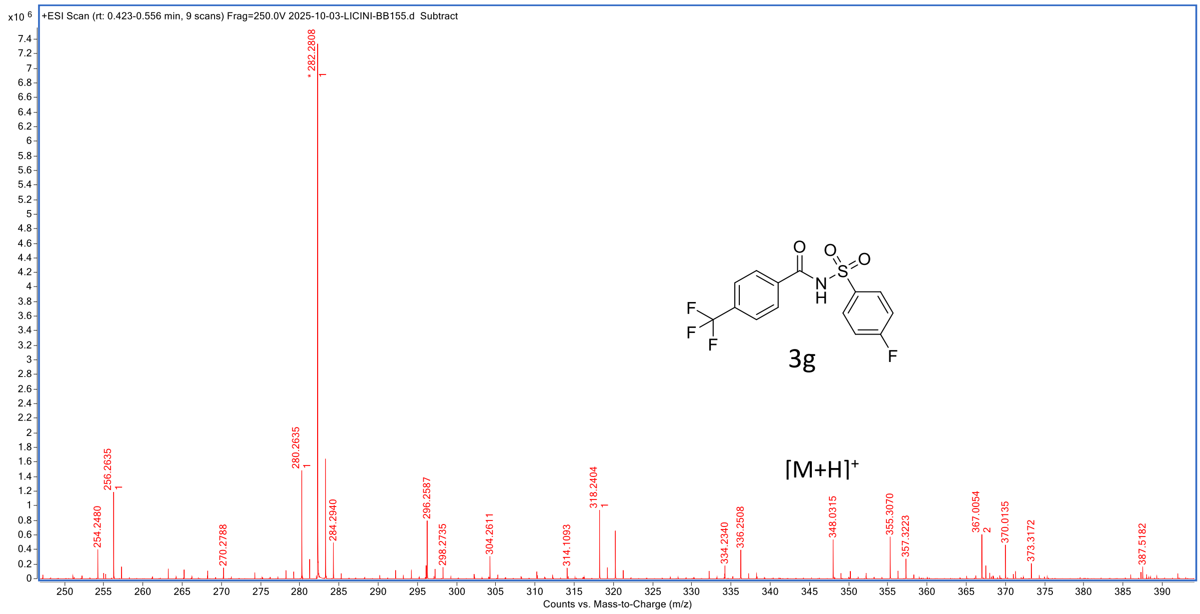

x10 6
+ESI Scan (rt: 0.423-0.556 min, 9 scans) Frag=250.0V 2025-10-03-LICINI-BB155.d Subtract
282.2808
280.2635
256.2635
254.2480
270.2788
284.2940
296.2587
298.2735
304.2611
314.1093
318.2404
334.2340
336.2508
348.0315
355.3070
357.3223
367.0054
370.0135
373.3172
387.5182
[M+H]+
3g
Counts vs. Mass-to-Charge (m/z)

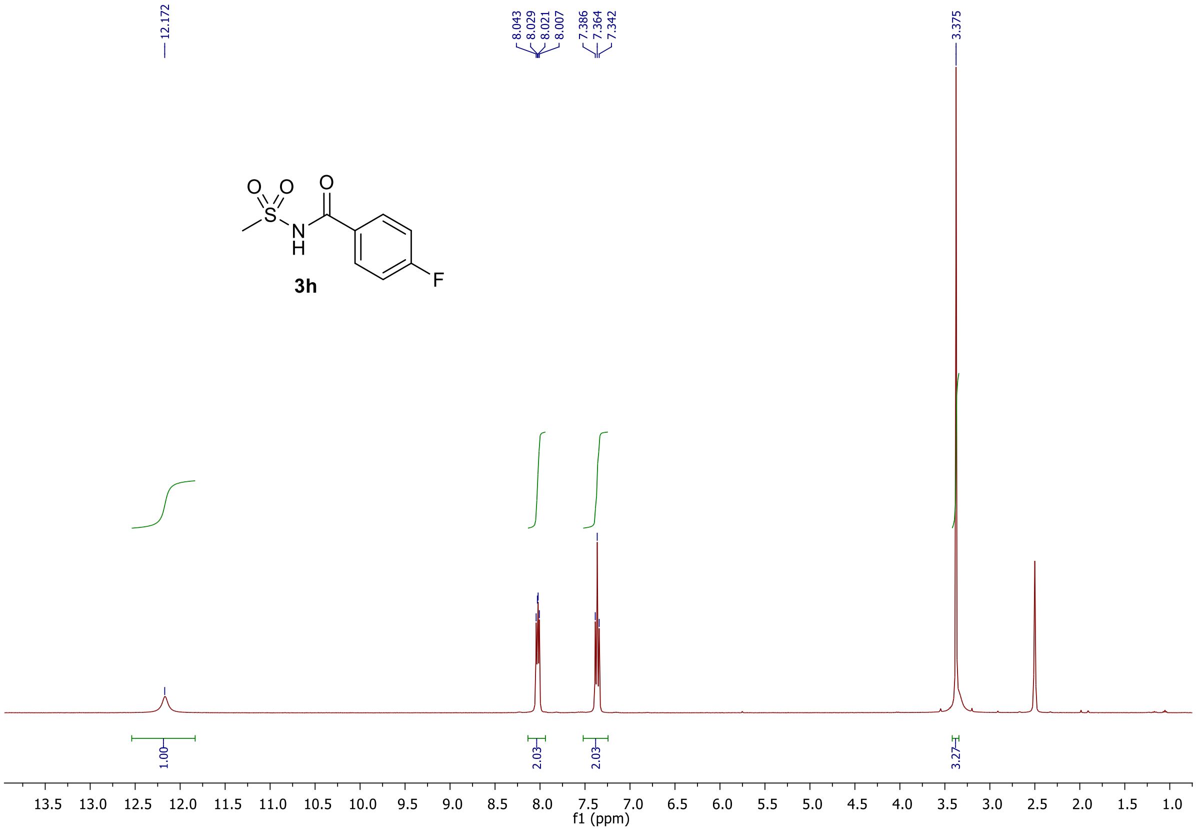

12.172
8.043
8.029
8.021
8.007
7.386
7.364
7.342
3.375
3h
1.00
2.03
2.03
3.27
f1 (ppm)

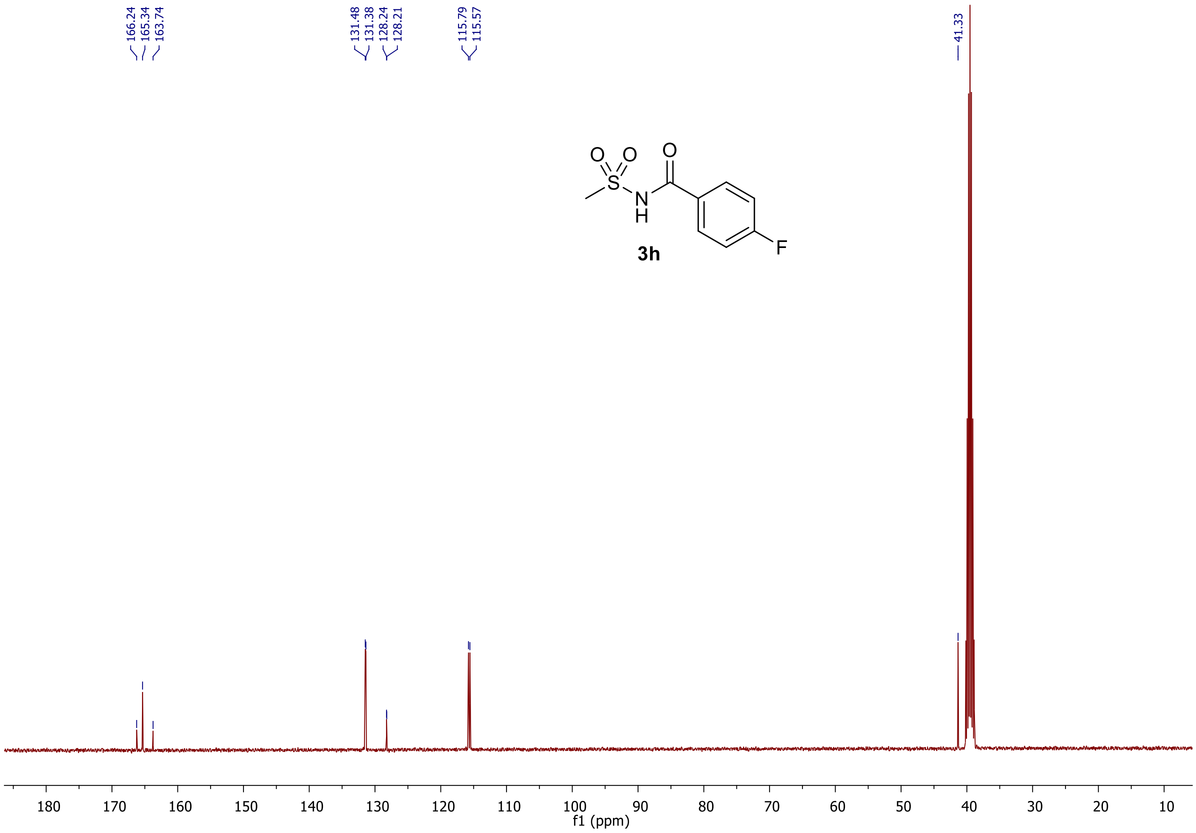

166.24
165.34
163.74
131.48
131.38
128.24
128.21
115.79
115.57
41.33
3h
180
170
160
150
140
130
120
110
100
90
80
70
60
50
40
30
20
10
f1 (ppm)

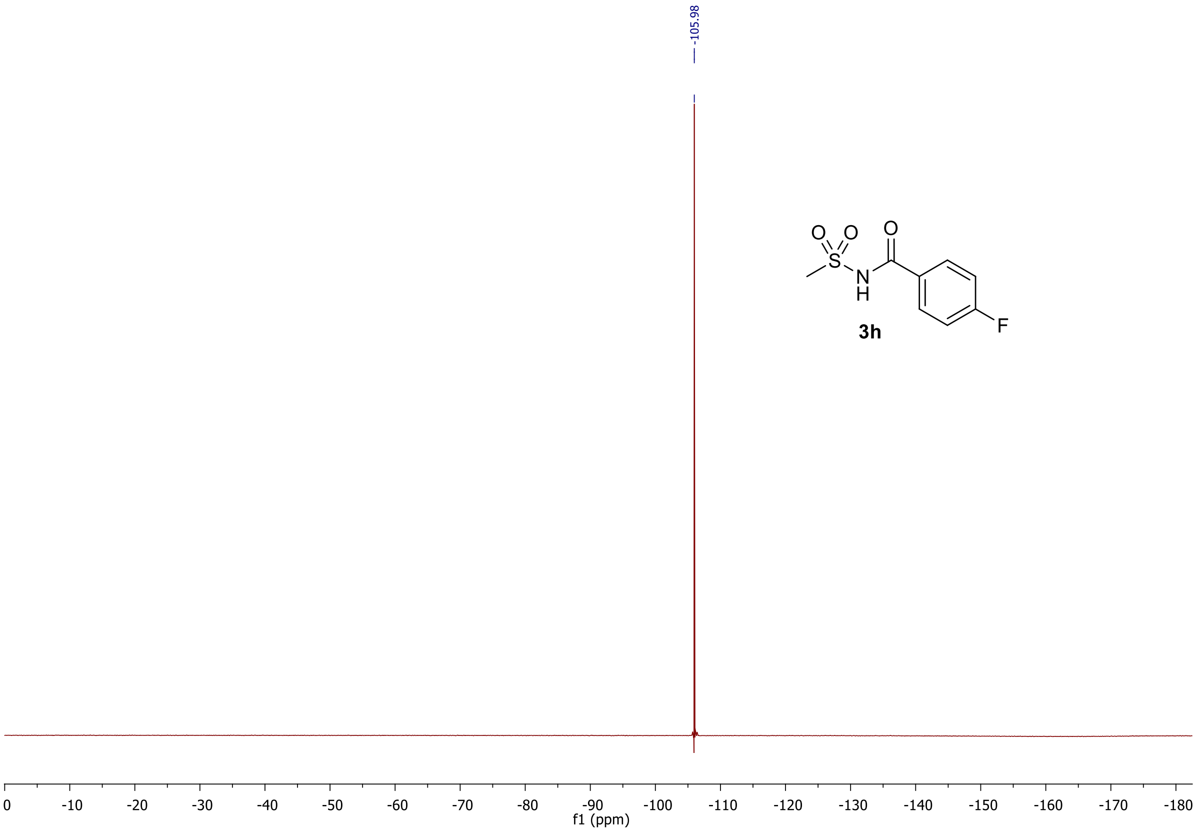

-105.98
3h
0
-10
-20
-30
-40
-50
-60
-70
-80
-90
-100
-110
-120
-130
-140
-150
-160
-170
-180
f1 (ppm)

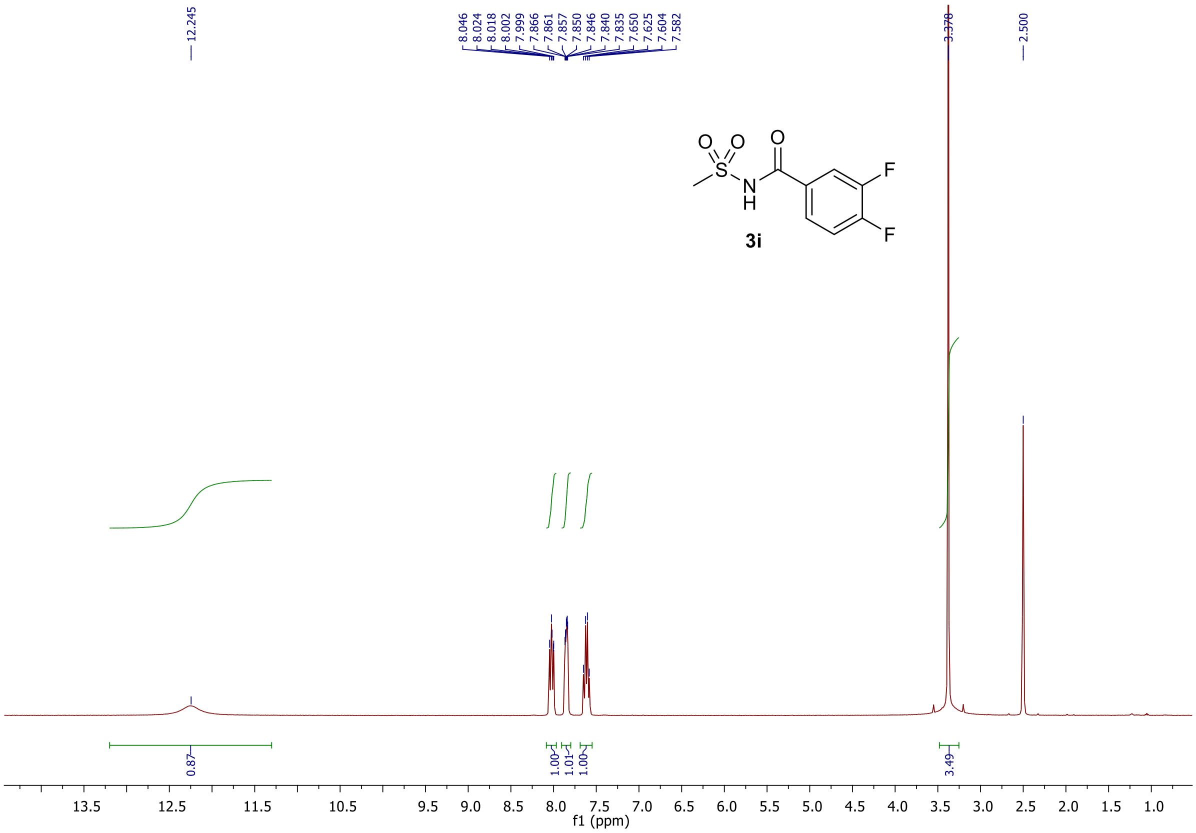
12.245
8.046
8.024
8.018
8.002
7.999
7.866
7.861
7.857
7.850
7.846
7.840
7.835
7.650
7.625
7.604
7.582
3.378
2.500
3i
0.87
1.00
1.01
1.00
3.49
f1 (ppm)

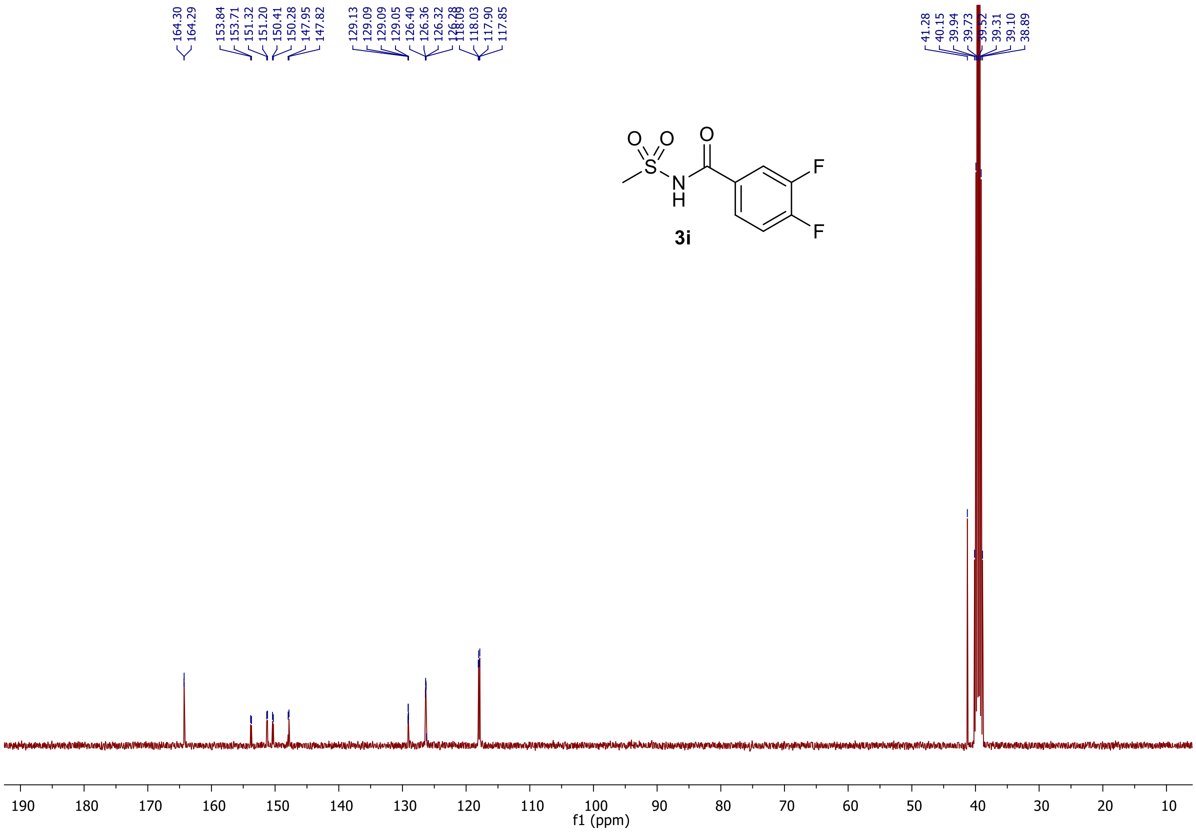
164.30
164.29
153.84
153.71
151.32
151.20
150.41
150.28
147.95
147.82
129.13
129.09
129.09
129.05
126.40
126.36
126.32
118.03
117.90
117.85
41.28
40.15
39.94
39.73
39.52
39.31
39.10
38.89
3i
f1 (ppm)

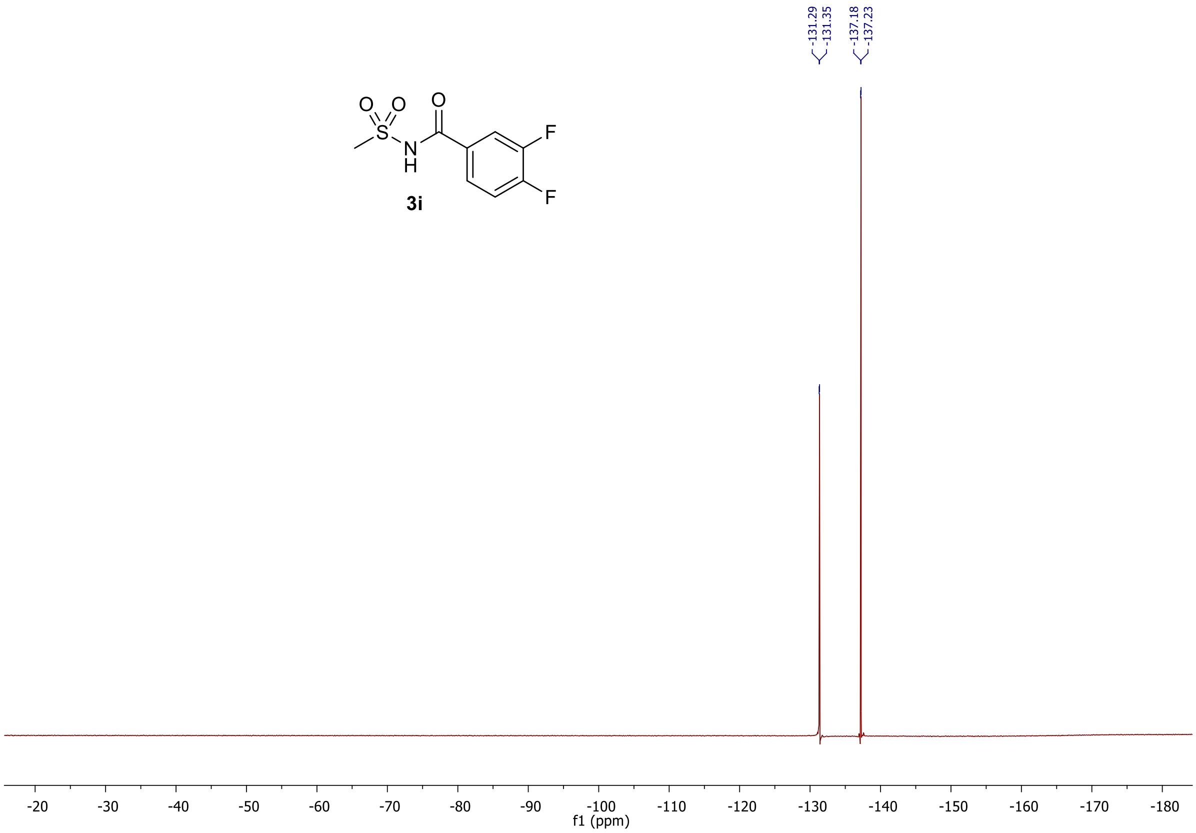
-131.29
-131.35
-137.18
-137.23
3i
-20 -30 -40 -50 -60 -70 -80 -90 -100 -110 -120 -130 -140 -150 -160 -170 -180
f1 (ppm)

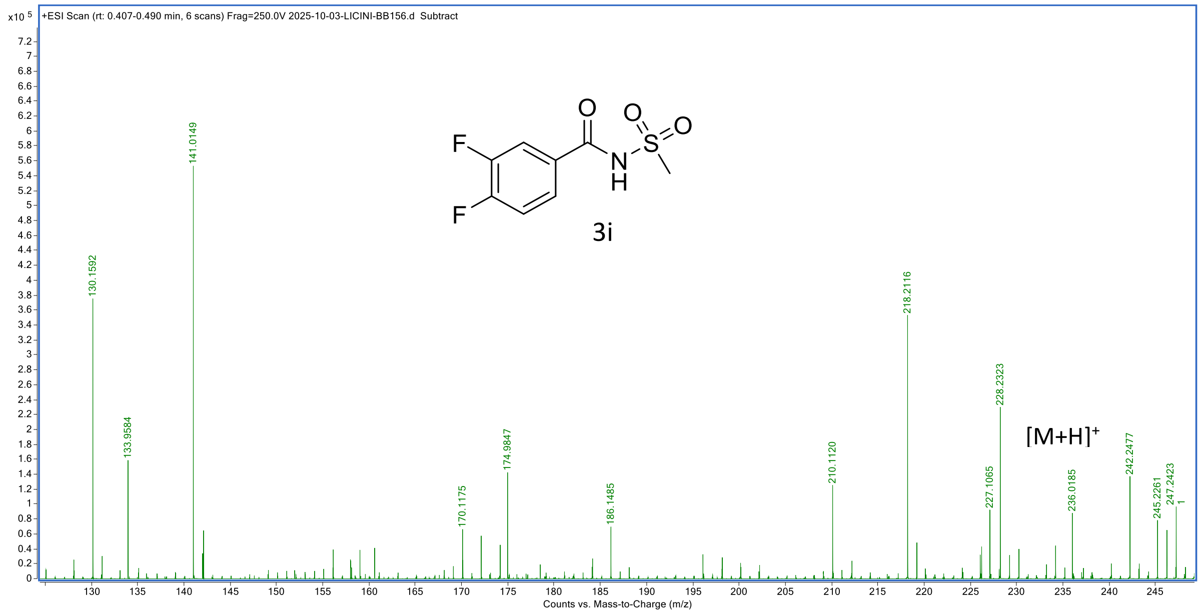
x10 5
+ESI Scan (rt: 0.407-0.490 min, 6 scans) Frag=250.0V 2025-10-03-LICINI-BB156.d Subtract
3i
[M+H]+
130.1592
133.9584
141.0149
170.1175
174.9847
186.1485
210.1120
218.2116
227.1065
228.2323
236.0185
242.2477
245.2261
247.2423
Counts vs. Mass-to-Charge (m/z)

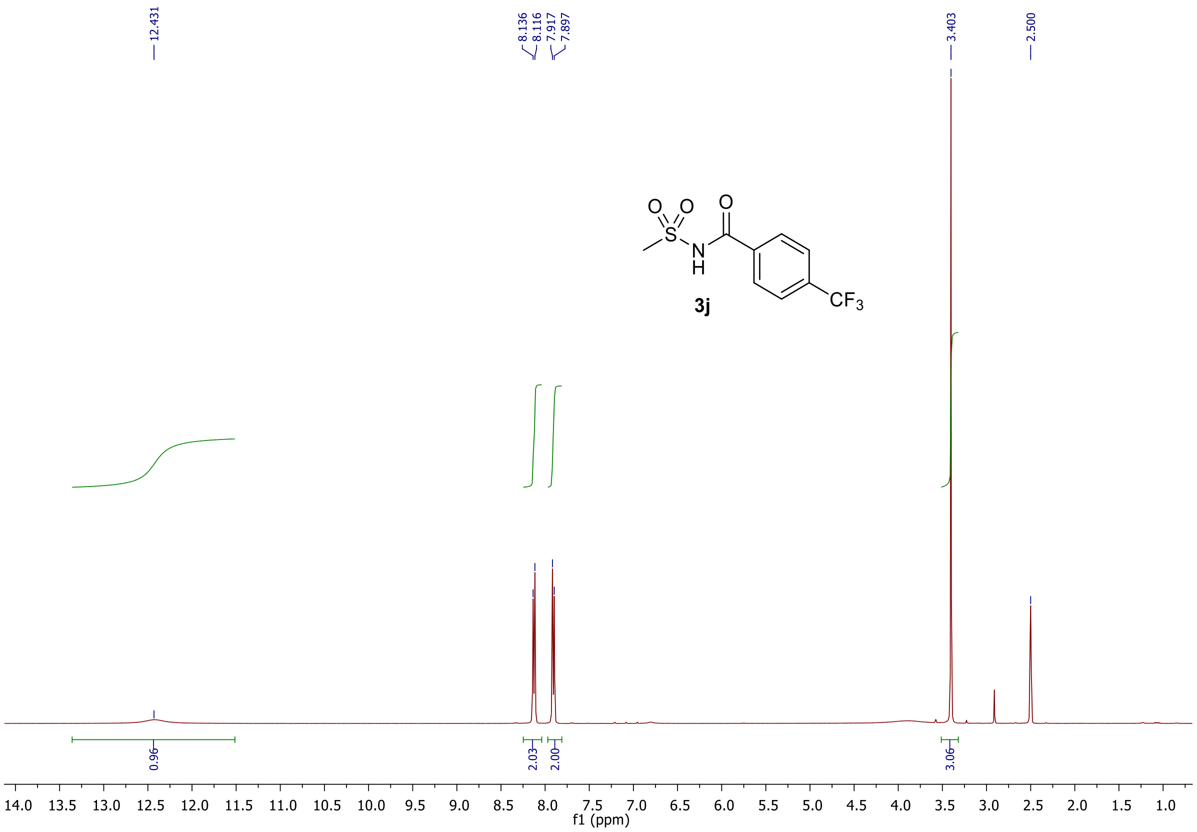
12.431
8.136
8.116
7.917
7.897
3.403
2.500
3j
0.96
2.03
2.00
3.06
14.0 13.5 13.0 12.5 12.0 11.5 11.0 10.5 10.0 9.5 9.0 8.5 8.0 7.5 7.0 6.5 6.0 5.5 5.0 4.5 4.0 3.5 3.0 2.5 2.0 1.5 1.0
f1 (ppm)

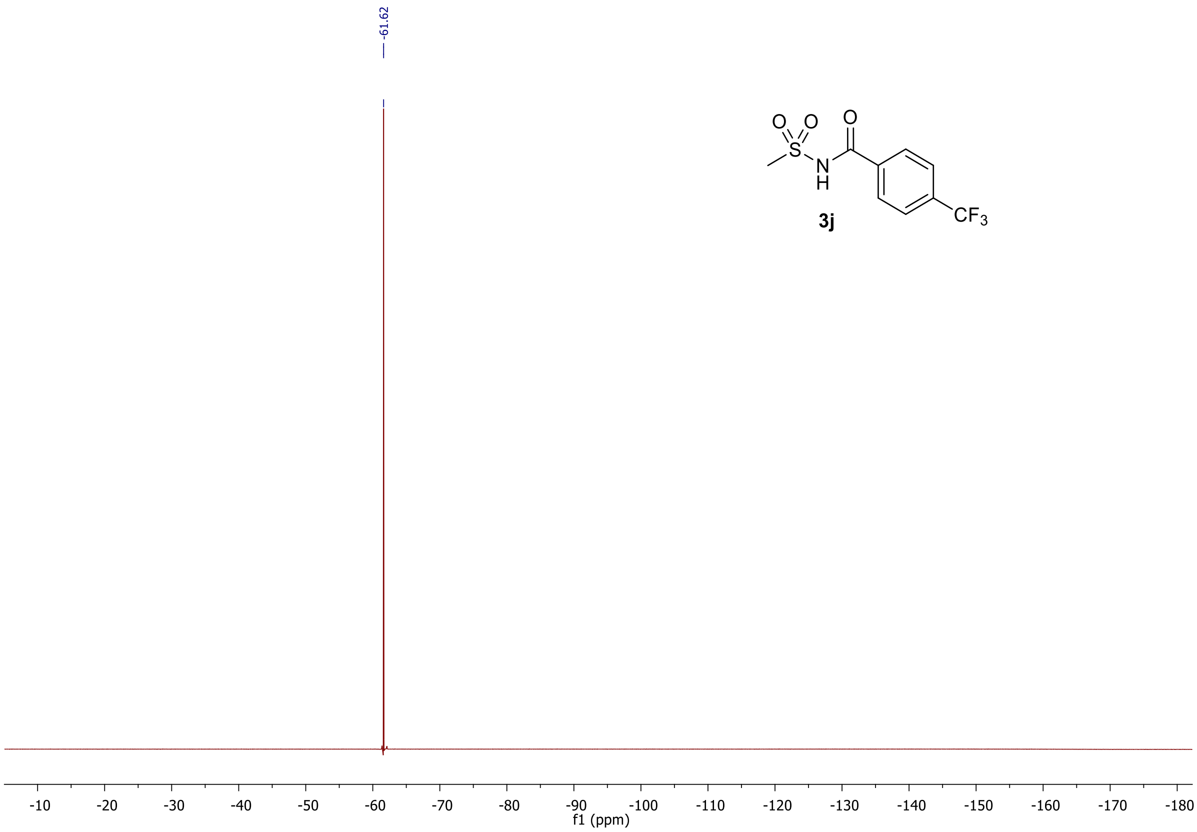
-61.62
3j
-10 -20 -30 -40 -50 -60 -70 -80 -90 -100 -110 -120 -130 -140 -150 -160 -170 -180
f1 (ppm)
